# The NRF2-CARM1 axis links glucose sensing to transcriptional and epigenetic regulation of the pentose phosphate pathway in gastric cancer

**DOI:** 10.1038/s41419-024-07052-3

**Published:** 2024-09-12

**Authors:** Miaomiao Ping, Guangyao Li, Qijiao Li, Yang Fang, Taotao Fan, Jing Wu, Ruiyi Zhang, Lesha Zhang, Bing Shen, Jizheng Guo

**Affiliations:** 1 School of Basic Medical Sciences, Anhui Medical University, Hefei, Anhui China; 2 Department of Gastrointestinal Surgery, The Second People’s Hospital of Wuhu, Wuhu, China; 3 Department of Otorhinolaryngology, Head and Neck Surgery, The First Affiliated Hospital of Anhui Medical University, Hefei, China

**Keywords:** Cancer metabolism, Cell signalling

## Abstract

Cancer cells autonomously alter metabolic pathways in response to dynamic nutrient conditions in the microenvironment to maintain cell survival and proliferation. A better understanding of these adaptive alterations may reveal the vulnerabilities of cancer cells. Here, we demonstrate that coactivator-associated arginine methyltransferase 1 (CARM1) is frequently overexpressed in gastric cancer and predicts poor prognosis of patients with this cancer. Gastric cancer cells sense a reduced extracellular glucose content, leading to activation of nuclear factor erythroid 2-related factor 2 (NRF2). Subsequently, NRF2 mediates the classic antioxidant pathway to eliminate the accumulation of reactive oxygen species induced by low glucose. We found that NRF2 binds to the CARM1 promoter, upregulating its expression and triggering CARM1-mediated hypermethylation of histone H3 methylated at R arginine 17 (H3R17me2) in the glucose-6-phosphate dehydrogenase gene body. The upregulation of this dehydrogenase, driven by the H3R17me2 modification, redirects glucose carbon flux toward the pentose phosphate pathway. This redirection contributes to nucleotide synthesis (yielding nucleotide precursors, such as ribose-5-phosphate) and redox homeostasis and ultimately facilitates cancer cell survival and growth. NRF2 or CARM1 knockdown results in decreased H3R17me2a accompanied by the reduction of glucose-6-phosphate dehydrogenase under low glucose conditions. Collectively, this study reveals a significant role of CARM1 in regulating the tumor metabolic switch and identifies CARM1 as a potential therapeutic target for gastric cancer treatment.

## Introduction

A limited supply of nutrients, particularly glucose, poses a huge obstacle for the survival of solid tumors [1, 2], but metabolic rewriting, an essential hallmark of cancer cells, enables them to optimize the utilization of scarce nutrients [3, 4]. The high adaptability and flexibility of cancer cells in nutrient acquisition and utilization sustain their survival and growth in nutrient-poor environments [5, 6]. The metabolic dependencies of cancer cells are distinct from those of normal cells and also serve as the “Achilles’ heel” of tumor cells [1, 7].

As a crucial component of cancer cell metabolism, glucose metabolism contributes essential intermediates and precursors to other vital metabolic pathways, including the synthesis of nucleotides, lipids, and amino acids [8, 9]. The pentose phosphate pathway (PPP) is a major glucose catabolic pathway that branches from glycolysis and is composed of an oxidative branch and nonoxidative branch [10]. The oxidative branch comprises three irreversible reactions that convert glycolytic intermediates, including the conversion of glucose-6-phosphate (G6P) into ribose-5-phosphate (R5P), and generate NADPH [10]. R5P is primarily used for de novo nucleotide biosynthesis. NADPH provides reducing power and is utilized for numerous anabolic reactions and antioxidation defense, which are necessary for cell survival and growth [8, 11]. The nonoxidative PPP branch consists of a series of reversible reactions that regulate the carbon flux between glycolysis and the PPP to meet the metabolic demands of cells [10, 12]. Emerging evidence indicates that PPP flux is enhanced and closely associated with the progression and drug resistance of many human cancers [13–16]. In cancer cells, PPP activity is tightly controlled by distinct mechanisms under certain stress conditions owing to the high levels of heterogeneity in various types of human cancers [9, 17]. However, how cancer cells coordinate glucose flux through sensing environmental glucose remains incompletely understood.

Coactivator-associated arginine methyltransferase 1 (CARM1, also known as PRMT4), is a member of the protein arginine methyltransferase family and plays a critical role in various cellular processes, including epigenetic regulation [18, 19], transcriptional regulation [20], chromatin remodeling [21], and DNA replication [18] by methylating histone H3 or non-histone proteins [22, 23]. Recent studies have elucidated the profound significance of CARM1 in the regulation of tumor metabolism [24, 25]. CARM1 interacts with and methylates pyruvate kinase M2 isoform (PKM2) to reprogram cancer metabolism from oxidative phosphorylation to aerobic glycolysis in breast cancer cells [26]. Alternatively, in mouse embryonic fibroblast cells, CARM1 methylates PKM2 to suppress glucose metabolism toward serine biosynthesis [27]. Chromosome 9 open reading frame 72 negatively regulates CARM1, which in turn regulates autophagy and lipid metabolism in mouse embryonic fibroblast under low glucose conditions [28]. However, the regulatory mechanisms of CARM1 expression and the role of CARM1 in regulating tumor metabolic pathways remain largely unclear.

Here, we report that nuclear factor erythroid 2-related factor 2 (NRF2) is capable of sensing glucose levels and directly upregulating the expression of CARM1. Subsequently, CARM1 mediates the methylation of H3R17, an epigenetic modification to the DNA packaging protein histone H3, leading to the positive regulation of G6PD expression, thereby promoting the PPP. These results reveal a non-canonical NRF2-CARM1 axis in the regulation of starvation-induced glucose metabolism and highlight the role of epigenetic dysregulation in cancer cell survival and proliferation under conditions of glucose deprivation.

## Materials and methods

### Antibodies and reagents

Antibodies against NRF2 (Abcam, ab62352, UK), CARM1 (Cell Signaling Technology, #12495, USA), β-actin (Proteintech, 66009-1-1g, USA), G6PD (Abcam, ab133525, UK), PGD (Santa Cruze, sc398977, USA), H3 (Cell Signaling Technology, #4499, USA), H3R17me2a (Abcam, ab8284, UK), and H3R26me2a (Abcam, ab194679, UK) as well as goat anti-rabbit horseradish peroxidase-conjugated secondary antibody (Elabscince Biotechnology, China) and goat anti-mouse horseradish peroxidase-conjugated secondary antibody (Elabscince Biotechnology, China) were purchased commercially.

NAC (Sigma, A7250, Shanghai, China), H_2_O_2_ solution (Sigma, 323381, Shanghai, China), H2DCF-DA (Sigma, 35845, Shanghai, China), 6-AN (Sigma, A68203, Shanghai, China), glucose (Hushi, 63005518, China), and glutamine (Gibco, 25030-081, USA) were also commercially obtained.

### Cell culture and treatment

All cell lines were purchased from the American Type Culture Collection or Procell (Wuhan, China). AGS cells were cultured in RPMI-1640 medium (Procell, PM550110, Wuhan, China) and HEK293T and MGC-803 cells were maintained in Dulbecco’s modified Eagle Medium supplemented with 10% fetal bovine serum (Biochannel, BC-SE-FBS007, Nanjing, China), 100 μ/ml penicillin G, and 100 μ/mL streptomycin at 37 °C in a humidified atmosphere containing 5% CO_2_. Plasmids were transfected either by using polyethylenimine or lipofectamine 3000 (Invitrogen, USA). Glucose-free medium was purchased commercially and supplemented with glucose of different concentrations and glutamine based on experimental needs. For N-acetyl-L-cysteine (NAC) supplement, cells were plated in culture medium with or without NAC pretreatment. The culture medium was changed 18 or 20 h before cell harvest. For H_2_O_2_ treatment, 1 mmol/L H_2_O_2_ was added to the culture medium 0.5 h before harvest.

### Human gastric tumor samples and IHC

A total of 16 surgical gastric cancer tissues and 16 adjacent non-tumor tissues were collected from the Second People’s Hospital of Wuhu, Department of Gastrointestinal Surgery. Written informed consent was obtained from each patient. The human study was approved by the Ethics Committee of the Second People’s Hospital of Wuhu, Anhui, China, and conducted in compliance with the relevant ethical regulations. Fresh gastric cancer and matched adjacent non-tumor tissues were immediately frozen in liquid nitrogen for subsequent Western blotting and IHC assays. IHC was performed as previously described [29]. To quantify the expression levels of CARM1, NRF2, G6PD, H3, and H3R17me2a, images were captured and analyzed using Motic Images Advanced software (version 3.0) by experienced pathologists. The mean density of the IHC images was determined by quantifying the intensity of the stained areas and expressing it as a ratio relative to the total area. This process involved the use of image analysis software. Initially, digital images of the IHC-stained sections were captured at a consistent magnification using a microscope. Subsequently, the software was utilized to set an appropriate threshold to distinguish the stained areas from the background, enabling the calculation of the percentage of stained area in relation to the total area.

### Western blotting

To extract proteins, cells were harvested and lysed in ice-cold NP-40 buffer (150 mmol/L NaCl, 50 mmol/L Tris-HCL, 0.3% NP40, pH 7.4) containing protease inhibitors (Beyotime Institute of Biotechnology, ST506, Shanghai, China) at 4 °C for 30 min. Standard western blotting protocols were employed to visualize protein levels. The band intensities of the Western blots were quantified using ImageJ software.

### Histone extraction

For histone extraction, cells were lysed in ice-cold HEPES buffer (10 mmol/L HEPES, 50 mmol/L NaCl, 0.5 mmol/L sucrose, 0.1 mmol/L EDTA, 0.5% Triton, pH 7.9) containing protease inhibitors. The nuclear fraction was obtained after centrifugation at 6500 × *g* for 10 min at 4 °C. After being washed twice with lysis buffer, the nuclear fraction was resuspended in 200 μL of 0.2 mol/L HCl at 4 °C overnight. To pellet the debris, the samples were centrifuged at 6500 × *g* for 10 min at 4 °C. The supernatant, which contained the histone protein, was collected and the remaining HCl was neutralized by the addition of 20 μL of 2 mol/L NaOH.

### Nuclear isolation

The isolation of nuclei was carried out utilizing a commercially available nuclei isolation kit (Sigma Aldrich, NUC201). In brief, adherent cells were thoroughly washed with phosphate-buffered saline (PBS) and subsequently scraped from the plate in the presence of lysis buffer. Cells were suspended in lysis media placed on top of a 1.8 M sucrose gradient. The resulting suspension underwent centrifugation at 30,000 × *g* for 45 min within a pre-cooled swinging bucket ultracentrifuge. Nuclei were collected as a white pellet at the bottom of the centrifuge tube and were subjected to further washing with the nuclei storage buffer provided with the kit. The isolated nuclei were promptly utilized for subsequent protein quantification.

### Generation of stable knockdown cell pools

CRISPR-Cas9 based gene knockout was performed with oligonucleotides, two sgRNAs containing the NRF2- and CARM1-targeting sequences, using the lentiCRISPR v2 system. The targeting sequences were as follows:

NRF2 sgRNA-1: 5′-TGGGACGGGAGTCCCGGCGG-3

NRF2 sgRNA-2: 5′-CCCGTCCCGGCACCACCGCA-3′

CARM1 sgRNA-1: 5′→3′ AACACCGACACGGTAGCGCA

CARM1 sgRNA-2: 5′→3′ CTCACCATCGGCGACGCGAA.

The sgRNAs targeting *NRF2* and *CARM1* were inserted into lentiCRISPR v2 vectors, and the two gastric cancer cell lines were transfected with plasmids using lipofectamine 3000 for 48 h. Cell selection was achieved by treatment with 1 μg/mL puromycin for 2 days to generate stable *NRF2* and *CARM1* knockdown cell pools. Whole cell lysates were collected, and NRF2 and CARM1 expression levels were detected use Western blotting.

### Measurement of intracellular ROS levels

ROS was quantified using chloromethyl-2′-7′-dichlorofluorescein diacetate (H_2_DCF-DA, Sigma, 35845), a fluorescent dye. Briefly, cells were seeded onto a 96-well plate and pretreated. Residual culture medium was removed, and a mixture of 3 μmol/L H_2_DCF-DA and fresh culture medium was added to the cells. The fluorescent dye was allowed to penetrate at 37 °C for 30 min. The cells were then washed twice with PBS, and the fluorescence intensity was measured at an excitation wavelength of 488 nm and an emission wavelength of 525 nm with a FlexStation 3 (Molecular Devices, USA).

### Dual-luciferase reporter assays

A 2-kilobase pair fragment from the promoter of CARM1 containing the putative binding sequences (wild-type/mutants) for NRF2 was synthesized and cloned into the firefly luciferase pGL3-control vector. For the reporter assay, HEK293T cells were plated on a 24-well culture plate. The cells in each well were co-transfected with 0.5 μg of PGL3 vector, CARM1 promoter WT-PGL3 or CARM1 promoter Mut1/2-PGL3, 0.5 μg of NRF2-pCDNA 3.1 or empty vector, and 0.1 μg of pRL-TK vector using Lipofectamine 3000 according to the manufacturer’s instructions. Cell lysates were collected 24 h post transfection. The activity levels of firefly and Renilla luciferase were quantified using a dual-luciferase reporter assay system (Promega, USA). The luciferase activity was detected using BioTek Synergy HTX reader and normalized to the Renilla luciferase activity.

### RNA isolation and quantitative RT-PCR

Total RNA was extracted from cells using Sparkzol Reagent (Sparkjade, AC0101-B, China). 1 μg of RNA was reverse transcribed into cDNA using a reverse transcription kit. Gene specific primers were used in the presence of SYBR Green Mix (Sparkjade, AH0104-B, Shandong, China). The expression of the target gene was normalized to actin genes. Fold changes in the expression of each gene were determined by the comparative cycle threshold method, and data from three independent experiments were analyzed. For specific primer sequences, see support information Supplementary Table 1.

### ChIP-qPCR assay

The ChIP-qPCR assay was performed as previously described [29]. Briefly, parental MGC-803 cells and those with *CARM1* knockdown cultured with normal or low glucose medium were crosslinked with 1% formaldehyde for 10 min at room temperature. The cells were then lysed and sonicated using a Bioruptor, with a high-output power setting for 20 cycles (30 s ON and 30 s OFF). The solubilized chromatin was subjected to immunoprecipitation using ChIP-grade antibodies specific for H3R17me2a or for rabbit IgG as a negative control. Prior to immunoprecipitation, the antibodies were preincubated with protein A sepharose beads (Santa Cruz) overnight at 4 °C. The antibody-chromatin complexes were subsequently pulled down using protein A sepharose beads, followed by extensive washing with high salt, lithium chloride, and TE buffer. Elution of the complexes was performed, and crosslinking was reversed in a water bath at 65 °C for 4 h. Proteinase K digestion was then carried out for 1 h at 55 °C. The Chipped DNA was purified using a QIA quick PCR Purification Kit (Qiagen, 28106) and analyzed by qPCR with SYBR Green using an ABI-7500 system (Applied Biosystems) and the primers specified in Supplementary Table 2.

### Metabolite extraction and analysis by HPIC-MS/MS

Metabolite quantification was carried out using high-performance ion exchange chromatography-tandem mass spectrometry (HPIC-MS/MS) with a system from Shanghai Biotree Biotech Co., Ltd. Cells were collected by trypsin digestion and counted. After centrifugation, the metabolite-extraction solution of 80% methanol was added to the sample. The samples were frozen with liquid nitrogen and thawed three times. Samples were centrifuged at 16,000 × *g* for 15 min at 4 °C to remove cell debris, proteins, and lipids. The supernatants were collected and subsequently transferred to inserts in injection vials for HPIC-MS/MS analysis. Electrospray ionization mass spectrometry in the negative mode was conducted using a SCIEX 6500 QTRAP^+^ triple quadrupole mass spectrometer. The final concentration (nmol·L^−1^) was determined by multiplying the calculated concentration (nmol·L^−1^) by the dilution factor. Metabolites were normalized to the cell number.

### NADPH and GSH/GSSG assays

NADPH levels were assessed utilizing a colorimetric NADP^+^/NADPH Quantitation Kit (Beyotime Institute of Biotechnology, Shanghai, China) following the manufacturer’s protocol. The absorbance at a wavelength of 450 nm was measured using a FlexStation 3 (Molecular Devices, USA) and normalized to the protein concentration. The ratio of GSH to GSSG was determined using commercially available kits (Beyotime Institute of Biotechnology, Shanghai, China). All procedures were carried out according to the instructions provided by the manufacturer.

### Cell proliferation assay and colony formation assay

The cells were subjected to trypsinization, resuspended in PBS, and then counted with a hemocytometer. For the cell proliferation assay, cells were seeded in 6-well plate at a density of 10,000 cells per well in 2 mL of culture medium containing 2.5 mmol/L glucose or H_2_O_2_ or 6-AN. The plates were counted at 2, 4, and 6 days. For colony formation assay, cells were seeded in 6-well plates at a density of 3000 cells per well in 2 mL of culture medium containing 2.5 mmol/L glucose. The medium was changed every 2 days. After 10 days, colonies were fixed in 4% paraformaldehyde and stained with 0.2% crystal violet. Colonies consisting of more than 50 cells were counted.

### EdU incorporation assay

Cells were treated with EdU for 2 h and subsequently examined using the Click-iT EdU Alexa Fluor Imaging Kit (ThermoFisher Scientific, USA) following the guidelines provided by the manufacturer. The images were captured using fluorescence microscopy.

### Cell cycle analysis

Parental cells and cells with *CARM1* knockdown were digested using trypsin and suspended in 500 μL of PBS. Cold absolute ethanol, stored at −20 °C, was then added dropwise to the cell suspension, and the suspension was kept at −20 °C for at least 2 h. Prior to staining, the cells were washed twice with cold PBS and centrifuged at 4 °C, 3000 × *g* for 5 min. The supernatant was completely removed. PI staining solution was added and the cells were incubated at 4 °C for 30 min. Data acquisition was performed using flow cytometry. The percentages of cells in the G_0_/G_1_, S, and G_2_/M phases were counted using ModFit LT software (BD Biosciences).

### Apoptosis assay

Apoptosis analysis was conducted using the Annexin V-FITC/PI Apoptosis Detection Kit (Bestbio, BB4101, Shanghai, China) and flow cytometry. In brief, suspended cells were centrifuged, collected, and washed twice in PBS. The cells were then resuspended in 400 μL of 1× Binding Buffer at a concentration of 1 × 10^6^ cells per mL. Subsequently, 5 μL of Annexin V-FITC was added to the cell suspension, which was gently mixed and incubated for 20 min at 4 °C, protected from light. Afterward, 10 μL of PI was added to the solution, mixed, and further incubated for 5 min at 4 °C, away from light. Flow cytometry analysis was performed using a flow cytometer.

### Xenograft mouse model

The procedures related to animal subjects were approved by Ethics Committee of Anhui Medical University, Hefei, China. In total, 6 × 10^6^ parental and *CARM1* knockdown MGC-803 cells were injected subcutaneously into the flanks of athymic nude mice (5 weeks old). Tumor volume was recorded by caliper measurements using the formula (length [mm]) × (width [mm]) × (height [mm]) × (π/6). For 6-AN treatment, tumor-bearing mice were given intraperitoneal administration of PBS or 6-AN (5 mg kg^−1^, twice a week). Tumors were dissected and weighed at day 30. Tumor volumes derived from parental and *CARM1* knockdown MGC-803 cells were compared.

### Statistical analysis

All data shown represent the results obtained from at least three independent experiments and are presented as mean ± SEM. Comparisons of mean values were analyzed by Student’s *t*-tests or two-way analyses of variance. A two-sided *P*-value < 0.05 was considered statistically significant.

## Results

### Elevated CARM1 levels correlate with NRF2 hyperactivation and poor prognosis among patients with gastric cancer

**Fig. 1:**
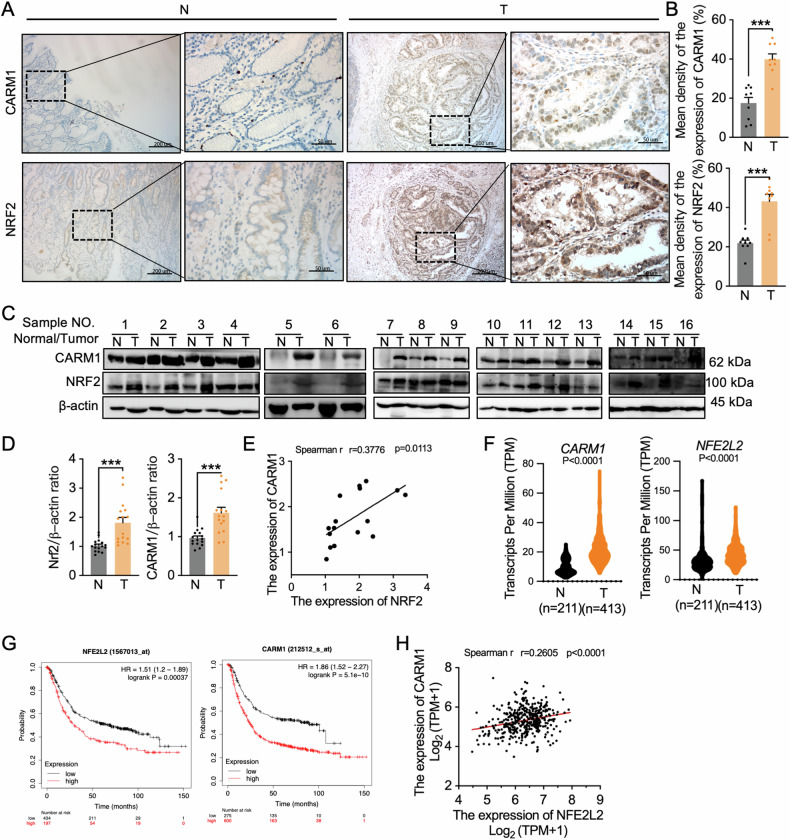
Elevated CARM1 levels correlate with NRF2 hyperactivation and poor prognosis of patients with gastric cancer. **A** Representative IHC images of CARM1 and NRF2 expression levels in specimens from 8 patients with gastric adenocarcinoma (T) and matched adjacent noncancerous tissues (N). Brown staining indicates the expression of the target protein as indicated. Scale bar, 200 μm. **B** CARM1 and NRF2 protein expression based on the staining index in gastric adenocarcinoma samples (*n* = 8) and normal adjacent tissues (*n* = 8). **C**, **D** Western blot analysis of CARM1 and NRF2 in gastric cancer tissues and matched adjacent noncancerous tissues. β-actin was used as a loading control. **E** Correlation between NRF2 and CARM1 protein expression levels in gastric cancer tissues. **F** Data from The Cancer Genome Atlas (TCGA) database showing CARM1 and NRF2 expression in adjacent (*n* = 211) and tumor tissues (*n* = 413) in patients with gastric cancer. **G** Kaplan–Meier survival curves with univariate analysis of patients with gastric adenocarcinoma based on high vs. low expression of CARM1 and NRF2. **H** Pearson correlation analysis of *NRF2* and *CARM1* gene expression in the TCGA dataset (*n* = 413). Data are shown as means ± standard errors of the mean (SEMs) with multiple replications. “*n*” indicates number of patients; *P < 0.05; ***P < 0.001.

Gastric cancer is a prevalent and highly lethal malignant neoplasm. Emerging evidence suggests that gastric cancer displays a diverse array of molecular aberrations encompassing a wide spectrum of epigenetic alterations [30]. To investigate the clinical relevance of CARM1 in gastric cancer, we examined the expression patterns of CARM1 in human gastric cancer tissues. As revealed by immunohistochemistry (IHC) staining, the expression level of CARM1 was significantly higher in gastric cancer tissues compared with adjacent noncancerous tissues (Fig. 1A, B). Similarly, increased expression of NRF2 was observed in gastric cancer tissues (Fig. 1A, B). Immunoblotting of gastric cancer sample tissues and their adjacent normal tissues demonstrated that CARM1 and NRF2 protein levels were significantly higher in gastric cancer tissue relative to surrounding normal tissue (Fig. 1C, D). Moreover, statistical analysis revealed a strong correlation between NRF2 and CARM1 at the protein level in gastric cancer tissues (Fig. 1E). Consistent with these findings, data from The Cancer Genome Atlas database showed that CARM1 and NRF2 were significantly upregulated in gastric cancer (Fig. 1F), and the higher expression levels of NRF2 and CARM1 were negatively correlated with the overall survival times of patients with gastric cancer (Fig. 1G). Moreover, Pearson correlation coefficient analysis indicated a positive correlation between NRF2 and CARM1 expression (Fig. 1H), suggesting that NRF2 may be involved in regulating the expression of CARM1.

### NRF2 senses glucose deprivation-induced reactive oxygen species (ROS) and directly upregulates the expression of CARM1

**Fig. 2:**
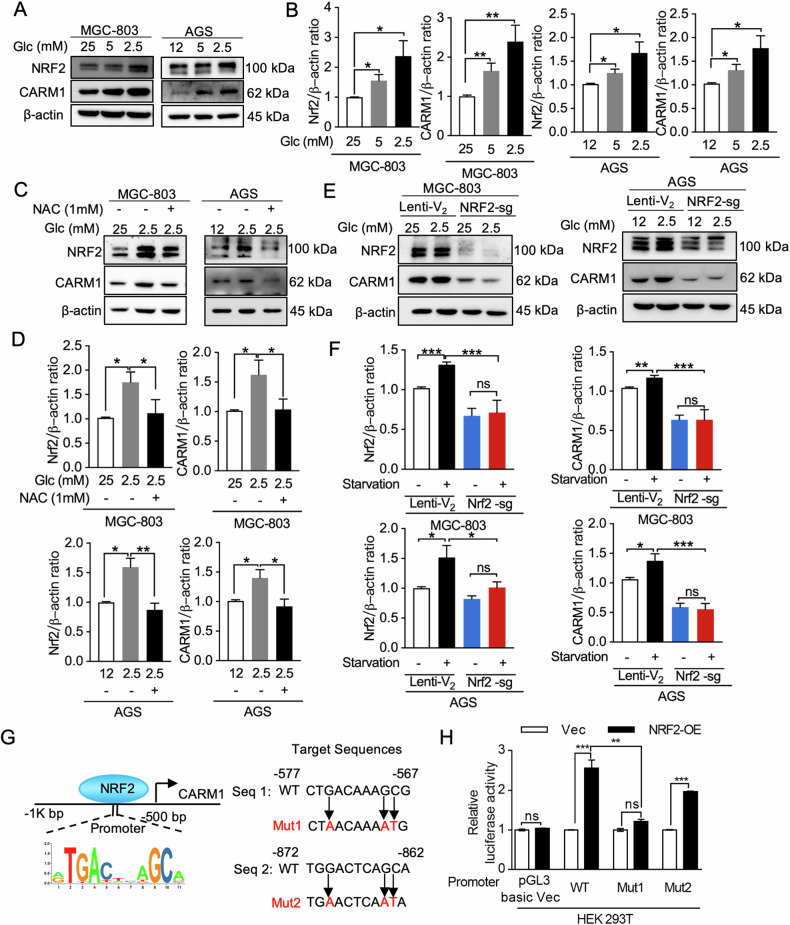
NRF2 senses glucose deprivation-induced ROS and directly upregulates the expression of CARM1. **A**, **B** MGC-803 and AGS cells were exposed to glucose deprivation as indicated for 18 h. Levels of CARM1 and NRF2 protein expression were analyzed by Western blotting. β-actin was used as a loading control. **C**, **D** MGC-803 and AGS cells were exposed to glucose deprivation and supplemented with or without 1 mmol/L NAC as indicated for 18 h, and the levels of CARM1 and NRF2 protein expression were determined. **E**, **F** Western blot images showing MGC-803 and AGS cells with parental NRF2 (Lenti-V2) or stable NRF2 knockdown (NRF2-sg) cultured with low glucose for 18 h (**E**). ImageJ was used to observe NRF2 and CARM1 protein expression (**F**). **G** Two predicted binding site sequences for NRF2 in the upstream promoter region of the CARM1 initiation sequence, along with information about the mutation positions, derived from the JASPAR database. **H** Dual-luciferase reporter assays were conducted using constructs containing predicted (WT) or mutated (Mut1, Mut2) target sequences to investigate the interaction between NRF2 and its target sequence. Data represent three independent experiments with triplicate measurements and are shown as mean ± SEM. Values of *P* < 0.05 were considered statistically significant. ns not significant; *P < 0.05, **P < 0.01, ***P < 0.001.

We next conducted experiments to determine whether CARM1 and NRF2 were regulated by metabolic signals. Several studies have shown that the glucose concentrations in human primary gastric cancer tissues are significantly lower than those in surrounding noncancerous tissues [31]. Consistent with our previous report [25], glucose starvation significantly increased intracellular ROS levels in gastric cancer cell lines MGC-803 and AGS (Fig. S1A). NRF2 plays a critical role in activating antioxidant defenses in response to oxidative stresses [15, 32]. As expected, glucose deprivation induced the activation of NRF2, thereby resulting in the upregulation of antioxidant genes *NQO1* and *GCLC* in mRNA and protein level (Fig. 2A, B and Fig. S1B, C and D). CARM1 was also upregulated in a dose-dependent manner in MGC-803 and AGS cells under conditions of glucose deprivation (Fig. 2A, B). Notably, glucose starvation upregulated NRF2 and CARM1, which was partially reversed by ROS scavenger NAC (Fig. 2C, D). Cellular ROS levels were measured after NAC treatment of cells under the same conditions (Fig. S2A). We simulated increased cellular ROS levels induced by low glucose using hydrogen peroxide (H_2_O_2_), and similar results were obtained (Fig. S2B, C). These results prompted us to speculate that glucose starvation-induced ROS may activate NRF2 to regulate the expression of CARM1. To further test whether protein levels of CARM1 were regulated by NRF2 following glucose starvation, we generated stable cells with knockdown of endogenous *NRF2* by using single guide RNA (sgRNA). As shown in Fig. 2E, F and Fig. S2B, C, knockdown of *NRF2* blocked glucose starvation and the H_2_O_2_-induced upregulation of CARM1. According to the JASPAR database, two potential NRF2 binding sites are predicted within the promoter region upstream of the CARM1 start sequence (Fig. 2G). Thus, we constructed luciferase reporter plasmids, one with a full-length promoter sequence (CARM1^P-WT^-Luc) inserted and another with two fragmented mutants (CARM1^P-Mut1/2^-Luc) containing potential binding sites upstream of the luciferase reporter gene (Fig. 2G). The results of the luciferase reporter assay indicated that activity levels of both the CARM1^P-WT^ and CARM1^P-Mut2^ promoter were enhanced after NRF2 overexpression, with no significant effect on the CARM1^P-Mut1^ promoter (Fig. 2H). These results indicated that NRF2 binding sites mapping to region 1 (−577 to −567), but not region 2 (−872 to −862), are required for NRF2-induced CARM1 transcription. Taken together, these data strongly suggest that glucose starvation increases CARM1 expression by activating NRF2 in a ROS-dependent manner.

### CARM1 enhances malignant behaviors of gastric cancer cells under low glucose conditions

**Fig. 3:**
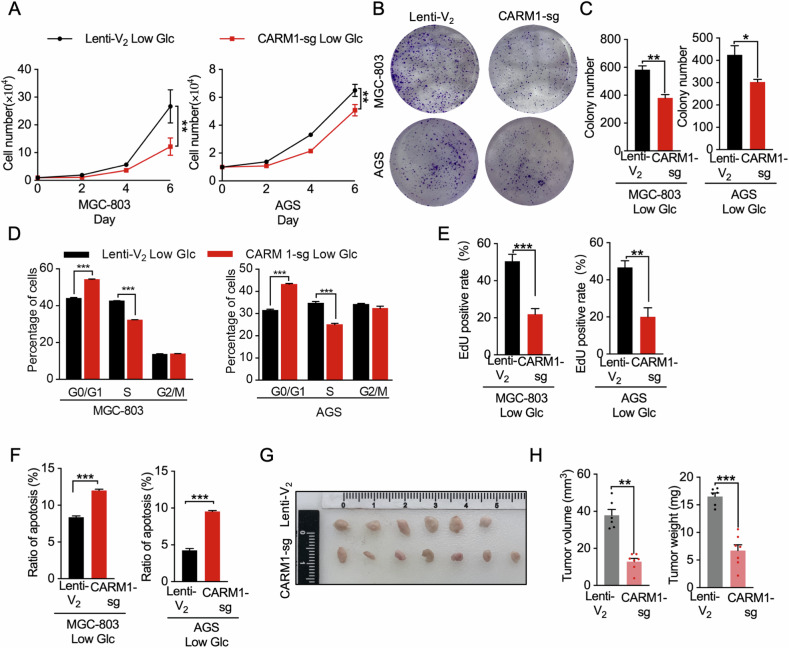
CARM1 enhances malignant behaviors of gastric cancer cells under low glucose condition. **A** Proliferation of MGC-803 and AGS cells with parental *CARM1* (Lenti-V_2_) or stable *CARM1* knockdown (CARM1-sg) treated with 2.5 mmol/L glucose (Low Glc) was determined by cell counting. **B**, **C** Colony formation assay of MGC-803 and AGS cells with Lenti-V_2_ or CARM1-sg treated with 2.5 mmol/L glucose. **D** MGC-803 and AGS cells with Lenti-V_2_ or CARM1-sg treated with 2.5 mmol/L glucose were harvested, fixed, and stained with propidium iodide for DNA content analysis using flow cytometry. Cell ModFit software was used to calculate the percentages of cells in the G1, S, and G2/M phases. **E** MGC-803 and AGS cells with Lenti-V_2_ or CARM1-sg were cultured in 6-well plates and treated with 2.5 mmol/L glucose for 18 h. Subsequently, the cells were exposed to EdU for 2 h and then stained with Apollo reaction cocktail for 30 min. Fluorescence microscopy was used to examine the EdU-labeled replicating cells. **F** Percentage of cells undergoing apoptosis, normalized against the total cell count in MGC-803 and AGS cells with Lenti-V_2_ or CARM1-sg under low glucose conditions. Cell apoptosis measured by Annexin-V/propidium iodide assays. **G**, **H** Dissected tumors from a xenograft mouse model with transplanted MGC-803 cells with Lenti-V_2_ or CARM1-sg (**G**). Volumes and weights of tumors on day 30 (**H**). Data are shown as mean ± SEM. Values of *P* < 0.05 considered statistically significant. *P < 0.05, **P < 0.01, ***P < 0.001.

The upregulation of CARM1 induced by glucose starvation suggests that it may play an important role in gastric cancer cells in response to low glucose stress. We hypothesized that CARM1 promotes cell survival and proliferation under low glucose conditions. In support of this theory, knockdown of *CARM1* significantly delayed the proliferation and colony formation ability of both MGC-803 and AGS cells under low glucose conditions (Fig. 3A–C). Flow cytometry analysis demonstrated that CARM1 depletion led to a significant increase in the percentage of cells in the G_0_/G_1_ phase and a decrease in the percentage of cells in the S phase peak (Fig. 3D), indicating a substantial inhibition of cell-cycle progression. We next explored whether CARM1 regulates nucleotide synthesis. In line with the cell cycle results, the 5-ethynyl-2′-deoxyuridine (EdU) incorporation rate was significantly lower in cells with the *CARM1* knockdown under low glucose conditions (Fig. 3E). However, in normal glucose conditions, the effect of *CARM1* deletion on the above-mentioned malignant behaviors of cells is significantly weaker than that in low-glucose culture (Fig. S3A–E). These results strongly indicate that CARM1 plays a more critical role in the proliferation and survival of gastric cancer cells under conditions of nutrient deficiency, highlighting its functional significance in low-glucose conditions.

Glucose deprivation significantly increased the intracellular ROS levels in MGC-803 and AGS cells, and *CARM1* knockdown led to a further increase of ROS levels in gastric cancer cells (Fig. S4A), indicating the involvement of CARM1 in the regulation of cellular ROS. Consistent with these results, glucose deprivation reduces the GSH/GSSG ratio and NADPH content in gastric cancer cells, and depletion of *CARM1* further causes a more pronounced decrease in the GSH/GSSG ratio and NADPH content (Fig. S4B, C).

ROS generation by glucose deprivation is intricately associated with the induction of apoptosis in cancers. Under low glucose conditions, cells with *CARM1* knockdown had a higher apoptotic rate compared with parental (*CARM1* wild type) cells (Figs. 3F, S4D). We further determined the viability of *CARM1* knockdown cells under oxidative stress conditions. Compared with parental cells, *CARM1* knockdown cells exhibited a lower tolerance in response to H_2_O_2_ (Fig. S4E). These data suggest that CARM1 is essential for protecting cancer cells from apoptosis and oxidative injury triggered by glucose starvation.

To further evaluate the effect of CARM1 on tumor growth, parental and *CARM1* knockdown AGS cells were injected subcutaneously into nude mice. The results showed that depletion of CARM1 resulted in the significant loss of their growth advantage when cells were grown in an adverse subcutaneous space in these mice (Fig. 3G, H).

### CARM1 drives carbon flux into the PPP by upregulating G6PD

**Fig. 4:**
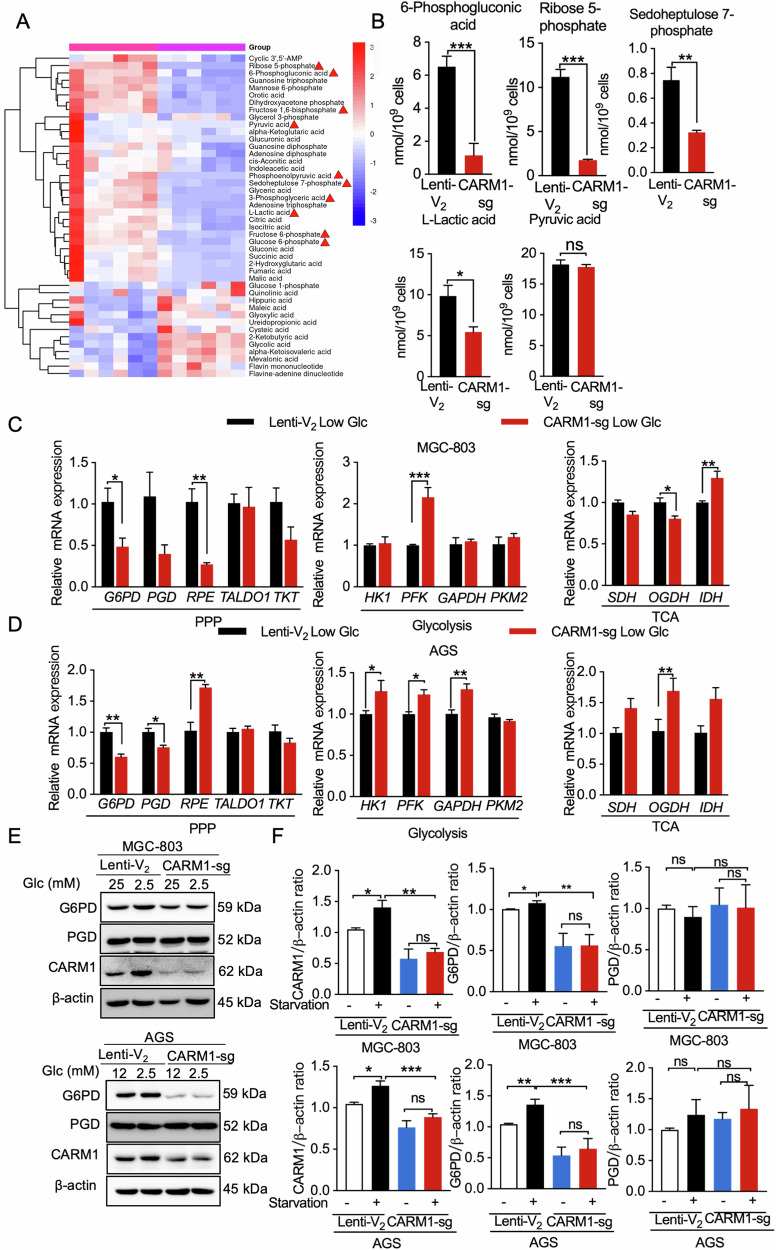
CARM1 drives carbon flux into the PPP by upregulating G6PD. **A** Quantitative proteomics was conducted to evaluate metabolites related to glucose metabolism. The heatmap displays the fold changes in central carbon metabolism in AGS cells with parental (Lenti-V_2_) or stable *CARM1* knockdown (CARM1-sg) under low glucose culture conditions. **B** Concentrations of significantly altered metabolites as analyzed by HPIC-MS/MS in samples extracted from AGS cells with Lenti-V_2_ or CARM1-sg treated with low glucose for 18 h. Results represent data from 6 independent experiments. **C**, **D** We used qPCR to determine expression levels of key enzymes involved in glucose metabolism in AGS cells with Lenti-V_2_ or CARM1-sg under low glucose (Low Glc) conditions. **E**, **F** Western blot analysis of MGC-803 and AGS cells with CARM1 knockdown treated with low glucose for 18 h (**E**). Summary data showing protein expression of G6PD and PGD obtained using ImageJ software (**F**). Data are shown as mean ± SEM with multiple replications. ns, not significant; *P < 0.05; **P < 0.01; ***P < 0.001.

Tumor cells have the ability to adapt metabolic pathways to optimize the utilization of nutrients and ensure cell survival and proliferation in response to nutrient deprivation [24, 26]. Whether elevated CARM1 is involved in the regulation of tumor cell glucose metabolic reprogramming is unclear. Therefore, we investigated the expression of enzymes in the main metabolic pathway of glucose through quantitative polymerase chain reaction (qPCR). We found that glucose starvation led to upregulation of CARM1 mRNA levels as well as upregulation of enzymes involved in glucose metabolism, including glucose transporter 1 (GLUT1), hexokinase 1, G6PD, and phosphogluconate dehydrogenase (PGD) (Fig. S5A, B). To further elucidate the role of CARM1 in regulating glucose metabolism, we performed liquid chromatography/high-resolution tandem mass spectrometry-based metabolomics on control and AGS cells with *CARM1* knockdown that were cultured under low glucose conditions. Targeted metabolomics analysis showed that knockdown of *CARM1* in gastric cancer cells under low glucose conditions resulted in significant changes of various metabolites (Fig. 4A). The levels of 6-phosphogluconic acid, R5P, and sedoheptulose 7-phosphate, metabolites in the PPP, were the most affected, decreasing below 40% in *CARM1*-silenced cells (Fig. 4B). This result indicated that there was a significant inhibition of the PPP in *CARM1* knockdown cells under low glucose conditions. In addition, *CARM1* knockdown also led to a significant suppression of lactate and ATP levels (Fig. S5C, D). The reduction in lactate production observed following *CARM1* knockdown may be attributed to the regulatory function of CARM1 in promoting the shift from oxidative phosphorylation to aerobic glycolysis [26, 33]. Alterations in the metabolite content of cells are often correlated with the levels and activity of their corresponding metabolic enzymes. Consistent with this correlation, *CARM1* knockdown significantly inhibited the upregulation of the *G6PD* and *PGD* genes induced by glucose starvation, but not other genes (Fig. 4C, D). Further investigation through immunoblotting indicated that glucose starvation induced an elevation in G6PD protein levels (Fig. 4E, F). Conversely, knockdown of *CARM1* suppressed the expression of G6PD and effectively abrogated its upregulation induced by glucose starvation. However, there was no significant alteration observed in the PGD protein expression level throughout the entire experiment (Fig. 4E, F). Similarly, *NRF2* knockdown blocked glucose starvation-induced upregulation of G6PD (Fig. S5E, F). Additionally, the intracellular ROS, GSH/GSSG, and NADPH, and other related substances content alterations induced by *NRF2* knockdown are in agreement with those resulting from *CARM1* knockdown. Thus, our results demonstrate that CARM1 upregulates the expression of G6PD by sensing the decline in extracellular glucose levels through NRF2, thereby eliciting activation of the PPP.

### G6PD is directly regulated by CARM1-mediated H3R17me2a

**Fig. 5:**
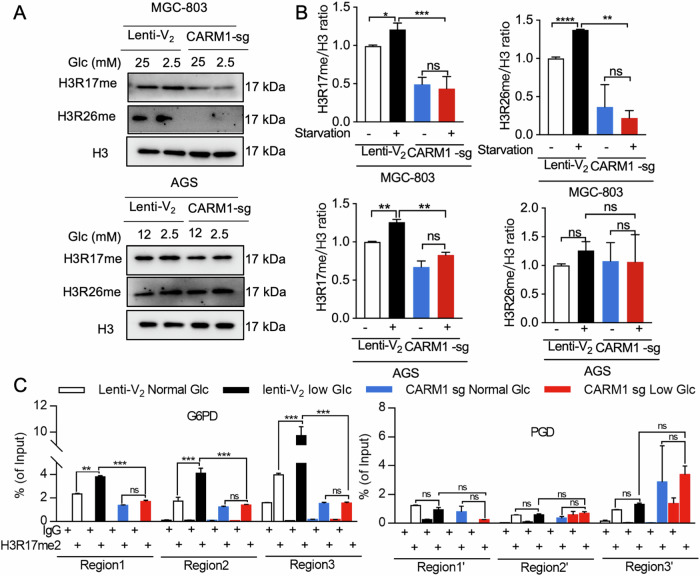
G6PD is directly regulated by CARM1-mediated H3R17me2a. Western blots showing H3R17 and H3R26 methylation levels of MGC-803 and AGS cells with parental CARM1 (Lenti-V2) and or stable CARM1 knockdown (CARM1-sg) that underwent glucose starvation for 18 h (**A**). Ratio of methylated H3R17 to H3R26 quantified using ImageJ (**B**). **C** MGC-803 cells with Lenti-V_2_ or CARM1-sg cultured in normal or low glucose (Glc) culture media for 18 h underwent ChIP-qPCR analysis for H3R17me2 at transcriptional start sites of *G6PD* and *PGD* genes. Data are shown as mean ± SEM with multiple replications. ns, not significant; *P < 0.05; **P < 0.01; ***P < 0.001.

As an epigenetic regulator, CARM1-mediated asymmetric dimethylation at arginine 17 and 26 of H3 (H3R17me2a/26me2a) has been implicated in the regulation of gene transcription [34, 35]. Specifically, methylation of histone H3 arginine 17 is associated with transcription activation [36]. Therefore, we hypothesized that G6PD was directly regulated by CARM1-catalyzed H3R17me2. In line with our previous findings, glucose starvation resulted in upregulation of CARM1 expression and concurrently resulted in marked upregulation of H3R17me2a levels, while no significant changes were observed in H3R26me2a levels (Fig. 5A, B). This finding may be attributed to the higher efficiency and preference of CARM1 for methylation of R17 compared with R26 [37]. Conversely, knockdown of *CARM1* suppressed the methylation of H3R17 and blocked its upregulation induced by glucose starvation (Fig. 5A, B). Consistent with this finding, the results of our nuclear-cytoplasmic fractionation experiments indicated that the upregulation of CARM1 induced by glucose starvation mainly occurs in the nucleus (Fig. S7A–C). It is noteworthy that the methylation levels of H3R26 exhibited inconsistent patterns in the two cell lines (Fig. 5A, B), suggesting that H3R26me2 may be cell type–dependent and may not be crucial for gastric cancer cells in response to glucose deprivation. Furthermore, the nuclear-cytoplasmic fractionation experiment showed that the increased CARM1 protein expression in the nucleus was consistent with that for the total protein (Fig. S7A). To assess the functions associated with H3R17 methylation, chromatin immunoprecipitation (ChIP) assays were performed on MGC-803 cells with antibodies against H3R17me2a. As shown in Fig. 5C, *G6PD*, but not *PGD*, was significantly occupied by H3R17me2a modifications around the transcriptional start sites, and the enrichment was increased when cells underwent glucose starvation. Notably, deletion of CARM1 reduced and blocked the increase of H3R17me2 on *G6PD*, but not on *PGD*. These data strongly support that glucose starvation-induced upregulation of CARM1 promotes the expression of G6PD by catalyzing the methylation of H3R17.

### Gastric cancer growth under low glucose conditions is dependent on CARM1-mediated PPP activity

**Fig. 6:**
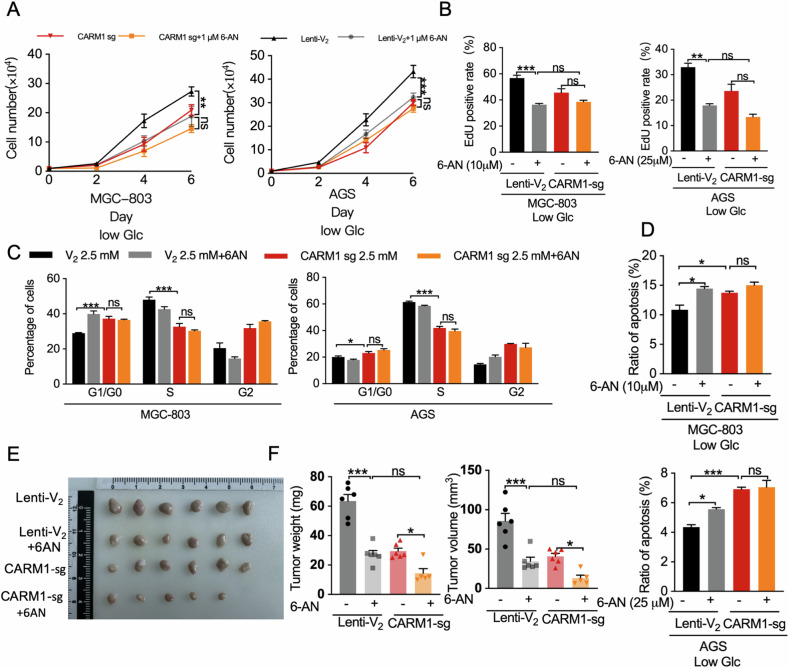
Gastric cancer growth under low glucose conditions is dependent on CARM1-mediated PPP activity. **A** Growth curves of AGS and MGC-803 cells with parental CARM1 (Lenti-V_2_) or stable CARM1 knockdown (CARM1-sg) were measured with or without 1 μM 6-AN treatment. **B** MGC-803 and AGS cells with Lenti-V_2_ or CARM1-sg were cultured in 6-well plates under the indicated conditions. Subsequently, the cells were exposed to EdU for 2 h and then stained with Apollo reaction cocktail for 30 min. Fluorescence microscopy was used to examine EdU-labeled replicating cells. **C** MGC-803 and AGS cells with Lenti-V_2_ or CARM1-sg treated with 2.5 mmol/L glucose in the presence or absence of 6-AN were harvested, fixed, and stained with propidium iodide for DNA content analysis using flow cytometry. Cell ModFit software was used to calculate the percentages of cells in the G1, S, and G2/M phases. **D** Apoptosis was measured by Annexin-V/propidium iodide assays for MGC-803 and AGS cells with Lenti-V_2_ or CARM1-sg under low glucose conditions with or without 6-AN as indicated. The percentage of cells undergoing apoptosis, normalized against the total cell count, was quantified. **E**, **F** MGC-803 cells with Lenti-V_2_ or CARM1-sg were inoculated into nude mice. Tumor-bearing mice were given intraperitoneal administration of PBS or 6-AN (dosage, 5 mg/kg, semiweekly). After 30 days, tumors were dissected from xenograft mouse models (**E**). Volumes and weights of the tumors (**F**). Data are shown as mean ± SEM. Values of *P* < 0.05 were considered statistically significant. ns, not significant; *P < 0.05, **P < 0.01, ***P < 0.001.

Given the observed function of CARM1 in cell growth and its effect on PPP activation, we speculated that gastric cancer cell proliferation and survival under low glucose conditions would rely on CARM1-mediated PPP activity. G6PD is the key rate-limiting enzyme that determines the magnitude of carbon flux into the PPP. Thus, we used 6-aminonicotinamide (6-AN) to blocked G6PD activity and the PPP [38, 39]. To facilitate a more comprehensive assessment of the impact of CARM1-regulated PPP regulation on the malignant behaviors of gastric cancer cells, we employed relatively high concentrations of 6-AN to ensure maximum inhibitory effects on the PPP. Similar to *CARM1* knockdown, 6-AN treatment effectively inhibited the proliferation of gastric cancer cells under low glucose conditions and was accompanied by substantial decreases in the levels of NADPH (Fig. S8A). Notably, the inhibitory effect of 6-AN on the proliferation of gastric cancer cells in *CARM1* knockdown cells showed no further reduction (Fig. 6A). Cell cycle and EdU assays yielded similar results (Fig. 6B, C). In addition, apoptosis analysis showed that treatment with 6-AN resulted in an increased proportion of cells undergoing apoptosis by blocking the PPP pathway (Fig. 6D). The inhibition of PPP by 6-AN did not exacerbate the apoptotic effects induced by *CARM1* knockdown (Fig. 6D). Taken together, these results suggested that the weakened cell proliferation and reduced anti-apoptotic effects following *CARM1* knockdown were mainly caused by the inhibition of the CARM1-mediated PPP activity.

We also tested the effects of 6-AN treatment on MGC-803 cells with wild-type *CARM1* or stable *CARM1* knockdown in xenograft models in vivo. As shown in Fig. 6E, F, 6-AN treatment significantly inhibited xenograft tumor growth with cells having wild-type *CARM1*, similar to the effects of CARM1 silencing. In addition, the inhibitory effect of 6-AN on xenograft tumors with CARM1 knocked down was attenuated. In line with these results, reduced G6PD and H3R17 methylation levels were observed in xenografts with *CARM1* knocked down (Fig. S8B). Collectively, the results indicate that the upregulation of CARM1 induced by glucose starvation supports cell growth and survival in gastric cancer cells through its positive regulation of the PPP.

### G6PD and H3R17me2 are upregulated in gastric cancer

**Fig. 7:**
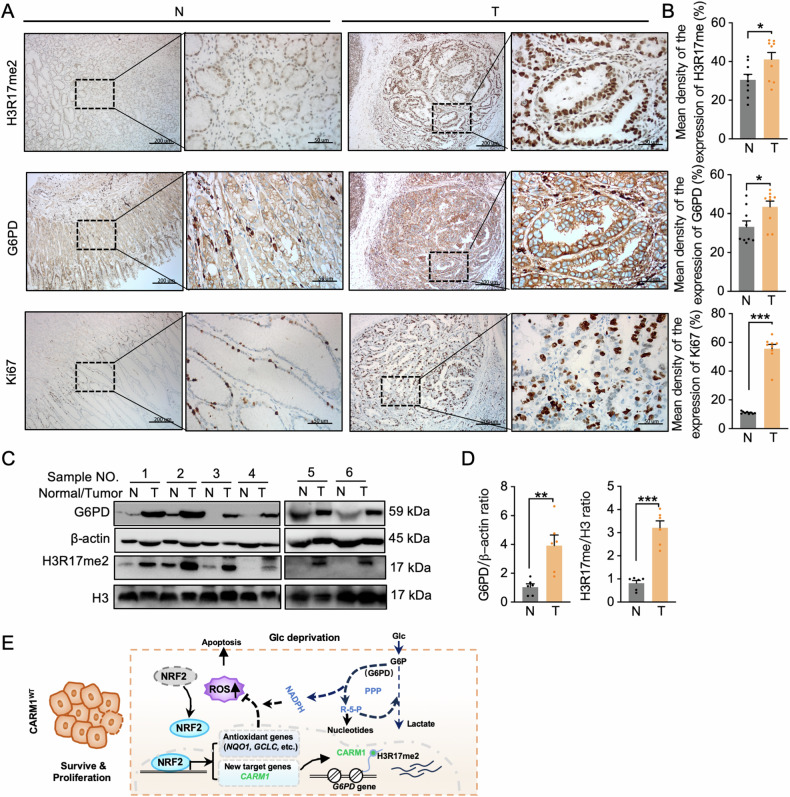
G6PD and H3R17me2 are upregulated in gastric cancer. **A** Representative IHC images of G6PD, H3R17me2, and Ki67 levels in specimens from 8 patients with stomach adenocarcinoma (T) and matched adjacent noncancerous (N) tissues. Brown staining indicates the expression of the indicated target protein. Scale bars, 200 μm. **B** G6PD and H3R17me2 protein expression levels based on the staining index in gastric adenocarcinoma samples (*n* = 8) and normal adjacent tissues (*n* = 8). **C**, **D** Western blot analysis of G6PD and H3R17me2 in gastric cancer tissues and matched adjacent noncancerous tissues. β-actin and histone H3 were used as loading controls in all immunoblots. **E** Working model for the NRF2-CARM1 axis enhancing the viability and proliferation of gastric cancer cells in glucose-deficient conditions by modulating the PPP. Glc represents glucose. All data are shown as mean ± SEM. *P* values were determined by two-tailed *t*-tests. Values of *P* < 0.05 were considered statistically significant. *P < 0.05, **P < 0.01, and ***P < 0.001.

To investigate the clinical relevance of our findings that G6PD was correlated with H3R17 methylation and was upregulated in gastric cancer cells under conditions of glucose starvation, IHC analyses were performed to examine G6PD expression and H3R17 methylation levels in human primary gastric cancer specimens. IHC staining (Fig. 7A, B) and a direct immunoblotting analysis (Fig. 7C, D) of gastric tumors and their adjacent normal tissues demonstrated that G6PD was overexpressed in gastric tumors, and methylation of H3R17 was at higher levels in tumor specimens than in normal control tissues. Consistent with our results shown in Fig. 1A, B, NRF2 hyperactivation, levels of CARM1, G6PD expression, and H3R17 methylation were significantly and positively correlated with each other. Together, these findings reveal a novel role for the NRF2-CARM1 axis in regulating the PPP in gastric cancer.

## Discussion

Metabolic rewiring serves as a defining characteristic of tumor cells, and it is critical for tumor cell survival and growth in response to certain genetic and environmental stressors [16, 40, 41]. Emerging evidence suggests that tumor cells induce metabolic reprogramming through a variety of regulatory pathways [42–44]. Although the mechanisms underpinning metabolic reprogramming are diverse, its main regulation involves the modulation of irreversible or rate-limiting enzymes in metabolic pathways, enabling efficient survival and proliferation under conditions of nutrient scarcity. In our study, we demonstrated that the upregulation of CARM1 in gastric cancer promotes glucose influx toward the PPP under metabolic stress by regulating the gatekeeper enzyme G6PD.

The PPP is a primary route for both glucose catabolism and biosynthesis [10, 45]. The “nature” of the PPP allows cells to effectively adapt to the metabolic demands under different conditions. We have observed that glucose deprivation leads to an accumulation of ROS within cells [25]. To maintain redox homeostasis, the PPP is modulated to enhance the oxidative branch to generated more NADPH, and the products are redirected through the non-oxidative branch to regenerate the glycolytic pathway metabolites, fructose-6-phosphate (F6P) and glyceraldehyde-3-phosphate (G3P), from pentose phosphate. Noteworthy, the entire process does not consume ATP, and we hypothesize that when ATP generation is limited, such as in the absence of glucose, this may be the most favorable metabolic pathway for the cell. Depending on the need, F6P is subsequently converted back to G6P to replenish the oxidative branch or direct flux into the later steps of glycolysis and oxidative phosphorylation [10, 46, 47]. Here, we showed that enhanced PPP activity induced by glucose deprivation promotes cell survival and counteracts oxidative stress by maintaining an adequate level of NADPH. Additionally, increased PPP activity can supply the substrate R5P for nucleic acid synthesis during cell proliferation. One limitation of our study is that we have not yet accurately measured the precise carbon flux ratio through the PPP and glycolysis under glucose starvation conditions.

CARM1, an arginine methyltransferase, is localized to both the nuclear and cytosolic compartments of cells [48]. It participates in the regulation of gene transcription activation and cellular metabolism through methylation of histone H3 and non-histone proteins [36, 49]. CARM1 is often overexpressed in several major cancer types, including ovarian, breast and colon cancers [50–52]. Currently, the specific mechanisms underlying the upregulation of CARM1 expression remain poorly understood, and CARM1 protein level regulation is mostly not at the transcriptional level [28, 53]. By contrast, our findings showed that NRF2 directly binds to the CARM1 promoter, modulating the expression of CARM1 at the transcriptional level. Previous studies have suggested that CARM1 has been identified as an oncogene, which regulates the malignant behaviors of cancer cells through various signaling pathways [24, 54, 55]. We discovered that CARM1 can modulate the glucose metabolic pathway in gastric cancer cells by enhancing the PPP to confront the stress of nutrient deprivation, consequently facilitating cell survival and proliferation. Moreover, in comparison to nutrient-rich conditions, CARM1 assumes a more crucial role for cells response to nutrient deprivation stress.

As an arginine methyltransferase, CARM1 is associated with the methylation of arginine residues on histones, particularly H3R17 and H3R26, leading to transcription activation [22, 35, 52]. Here, we found that the upregulation of CARM1 induced by low glucose significantly increased the levels of H3R17me2a, but not H3R26me2a, on the *G6PD* promoter. A reasonable explanation for these results is that methylation at H3R17 by CARM1 is more efficient and preferred over methylation at H3R26 [37]. Many effector molecules, such as Tudor domain-containing protein TDRD3 and the PAF1 complex, have been identified as specifically recognizing and binding to modified H3R17me2a, thereby mediating transcriptional activation [36, 56]. As demonstrated in previous studies, NRF2 serves as a transcriptional factor that governs the expression of numerous genes, and G6DP has been identified as a direct downstream target of NRF2 [57, 58]. However, we discovered that when *CARM1* was knocked down, activation of NRF2 no longer led to an increase in G6DP expression, indicating that the direct regulation of G6PD expression by NRF2 also relies on CARM1. It is yet to be determined whether NRF2 is an effector molecule capable of interacting with H3R17me2a to stimulate G6PD transcription.

In conclusion, we demonstrated that NRF2 is activated in response to elevated intracellular levels of ROS induced by glucose deprivation. NRF2 upregulates the expression of antioxidant genes through the classic antioxidant pathway. Cells transmit a signal of glucose deprivation in the form of ROS through NRF2 to CARM1, leading to upregulation of CARM1. The upregulated CARM1 causes hypermethylation of H3R17, thereby upregulating G6PD and promoting PPP activity. The PPP-enhanced activity in a CARM1-dependent manner produces intermediate metabolites for ROS scavenging and nucleic acids, thereby facilitating the survival and growth of gastric cancer cells under metabolic stress (Fig. 7E). These findings have significant implications for the development of precise therapeutic approaches based on CARM1 for patients with gastric cancer.

## Supplementary information

Supplementary Figure

Supplementary table 1

Supplementary table 2

full length uncropped original western blots

## Data Availability

The data used to support the findings of this study are available from the corresponding author upon request.
