# Supplementary material for: The NRF2-CARM1 axis links glucose sensing to transcriptional and epigenetic regulation of the pentose phosphate pathway in gastric cancer
Source: Cell Death Dis. 2024 Sep 12;15(9):670. doi: 10.1038/s41419-024-07052-3 (PMC11393079; doi:10.1038/s41419-024-07052-3)
Supplement: Supplementary file 1 — Supplementary Figure [file 41419_2024_7052_MOESM1_ESM.docx]

**Supplementary information for**

**The NRF2-CARM1 axis links glucose sensing to transcriptional**

**and epigenetic regulation of the pentose phosphate pathway in gastric cancer**

Miaomiao Ping, *et al*.

**Supplementary Figure 1**


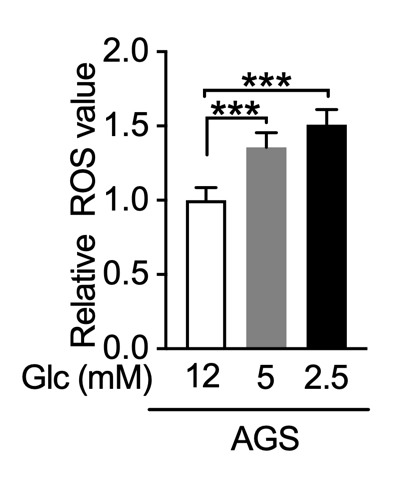

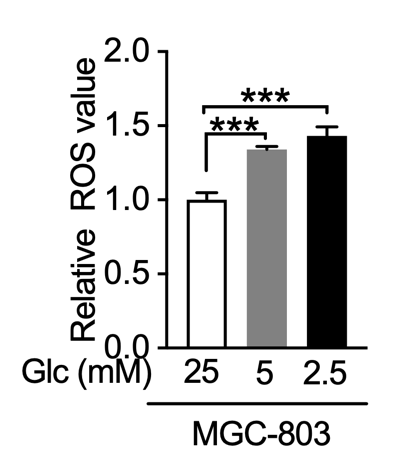

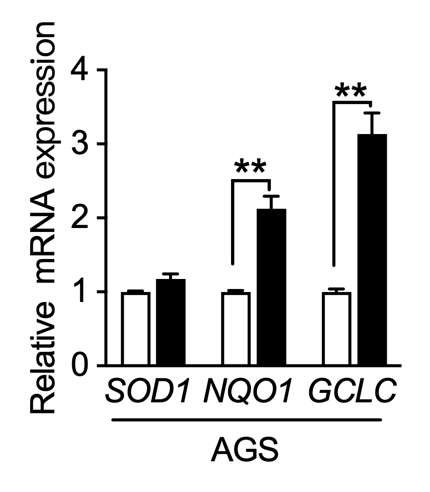

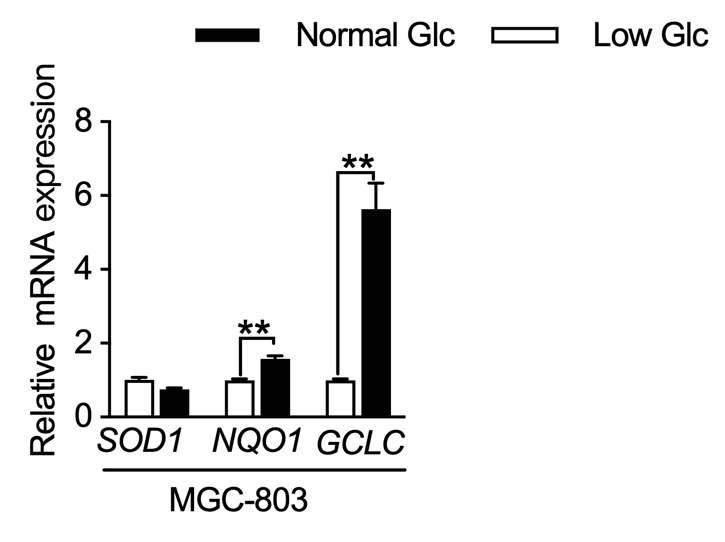


A

B


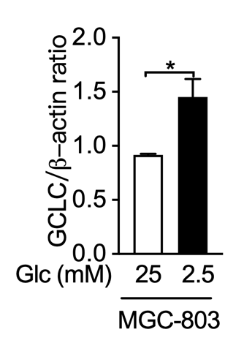

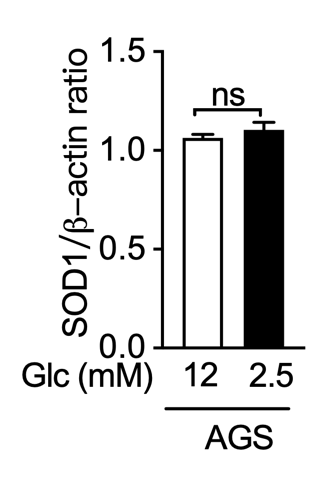

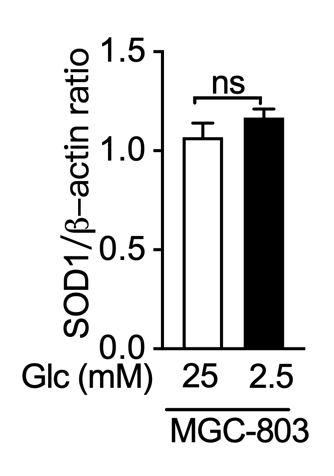

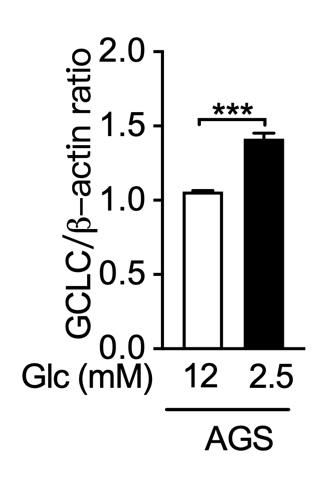

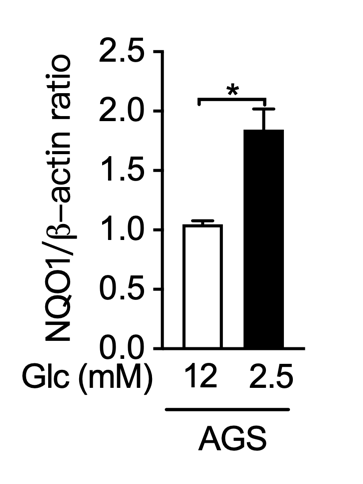

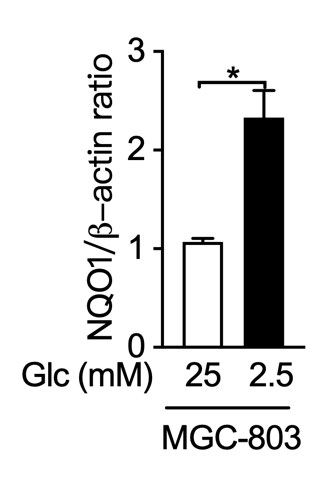


73 kDa

20 kDa

30 kDa

45 kDa


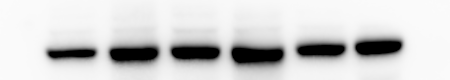


GCLC


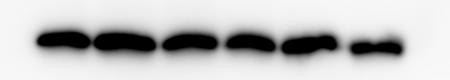


SOD1

Glc (mM)

25

2.5

25

2.5

25

2.5


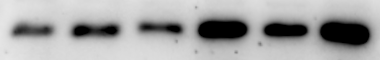

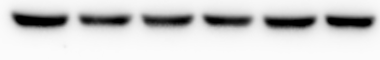


NQO1

β-actin

MGC-803


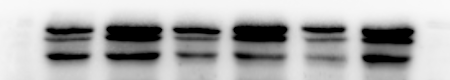

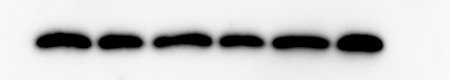


Glc (mM)


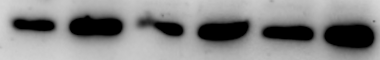

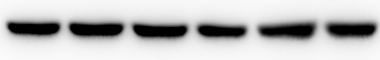


GCLC

SOD1

NQO1

β-actin

73 kDa

20 kDa

30 kDa

45 kDa

12

2.5

12

2.5

12

2.5

AGS

C

D

**Supplementary Figure 1. Glucose starvation results in cellular ROS accumulation and activation of NRF2.** A. Cellular ROS was measured in MGC-803 and AGS cells with the indicated concentration of glucose incubated for 18 h using the ROS-sensitive fluorescent dye CM-H_2_DCFDA and flaxstation3. B. Quantitative PCR analysis of the expression of NRF2 target genes in MGC-803 and AGS cells after glucose deprivation. C, D. MGC-803 and AGS cells were exposed to glucose deprivation as indicated for 18 h. Levels of GCLC, SOD1 and NQO1 protein expression were analyzed by Western blotting. β-actin was used as a loading control. All data are shown as mean±SEM. The P values were determined by two-tailed t-tests, and values of P<0.05 were considered statistically significant. ns, not significant; *, P<0.05; **, P<0.01; ***, P<0.001.

**Supplementary Figure 2**


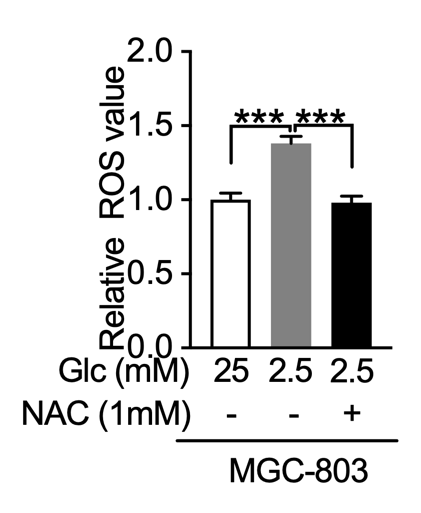

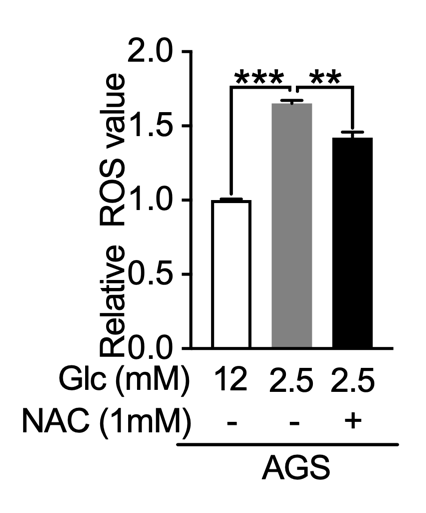


AGS

+

Lenti-V_2_

-

-

+

NRF2-sg


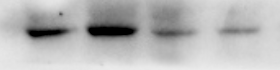

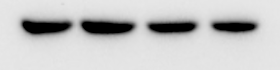

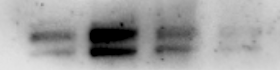


NRF2

CARM1

β-actin

H_2_O_2_ (1mM)

100 kDa

62 kDa

45 kDa


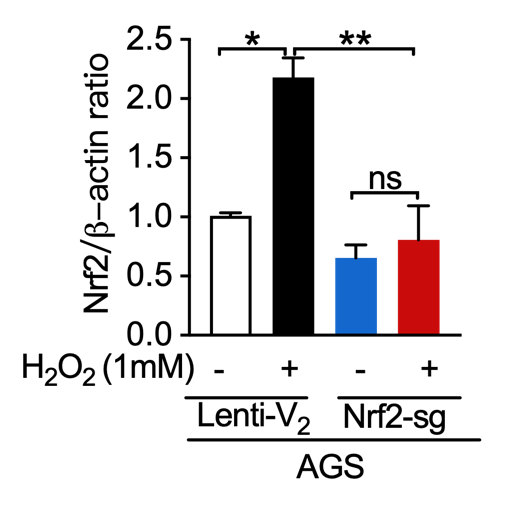

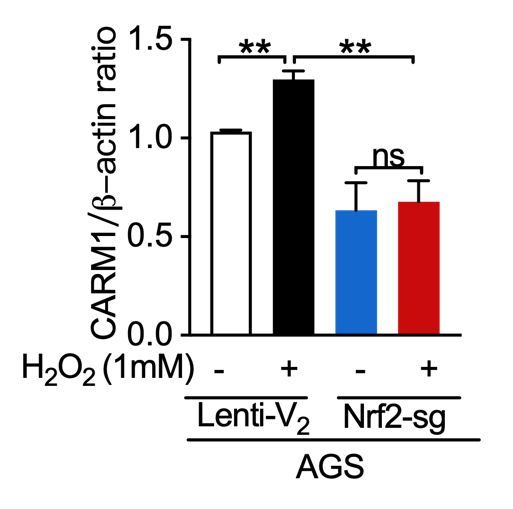

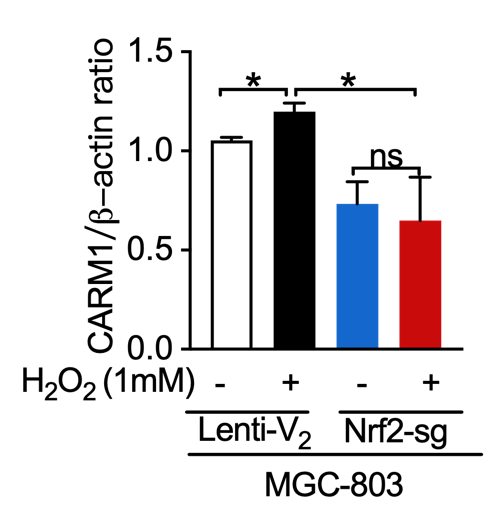

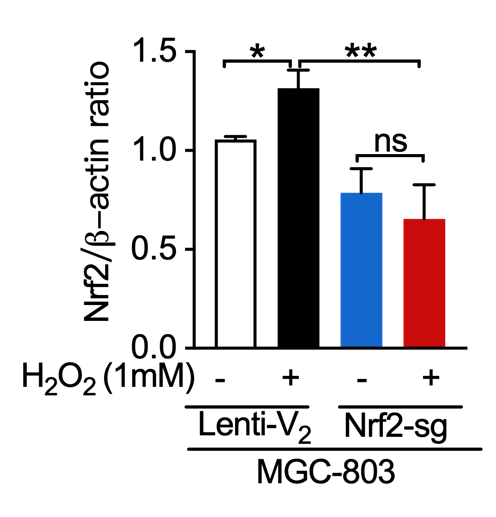


-

Lenti-V_2_

MGC-803

+

-

NRF2

CARM1

β-actin

+

H_2_O_2_ (1mM)

NRF2-sg

100 kDa

62 kDa

45 kDa


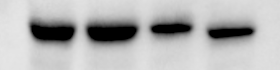

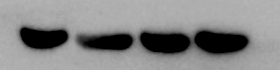


C

B

A


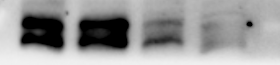


**Supplementary Figure 2. The upregulation of CARM1 induced by glucose starvation is dependent on ROS.** A. MGC-803 cells and AGS cells were treated with the indicated concentration of glucose and supplemented or not with 1 mM NAC for 18 h prior to measurement of ROS. B, C. CARM1 and NRF2 levels were analyzed in MGC-803 and AGS cells with parental *NRF2* (Lenti-V_2_) or *NRF2* knockdown (NRF2-sg) after treatment or not with 1 mM H_2_O_2_ for 0.5 h. β-actin served as the loading control. All data are shown as mean±SEM. The P values were determined by two-tailed t-tests, and values of P<0.05 were considered statistically significant. ns, not significant; *, P<0.05; **; P<0.01.

**Supplementary Figure 3**


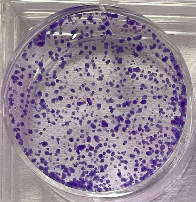

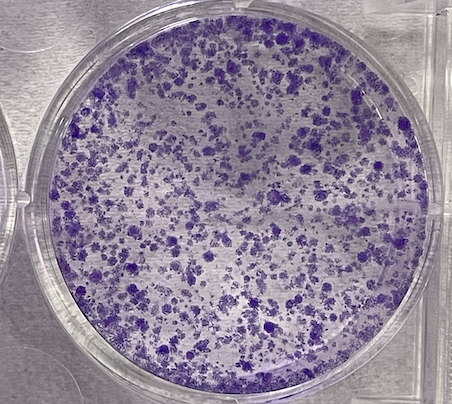

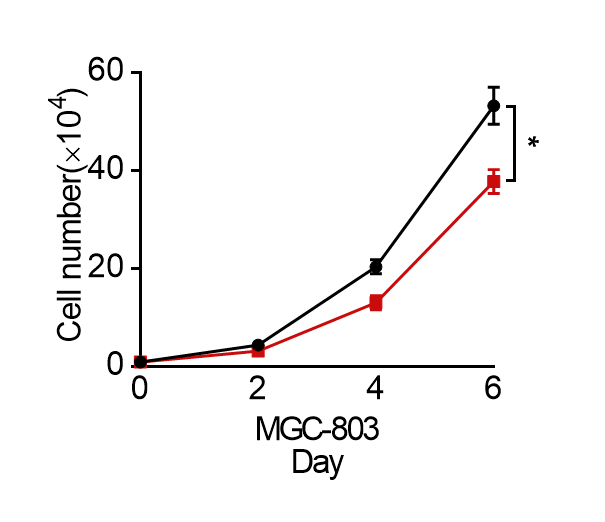

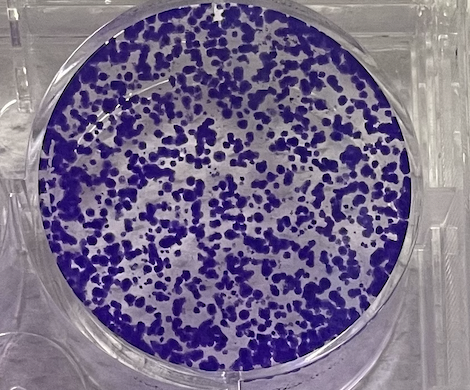

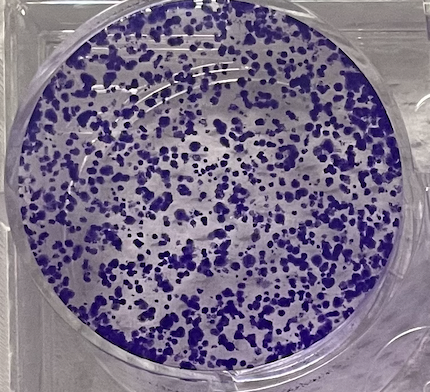

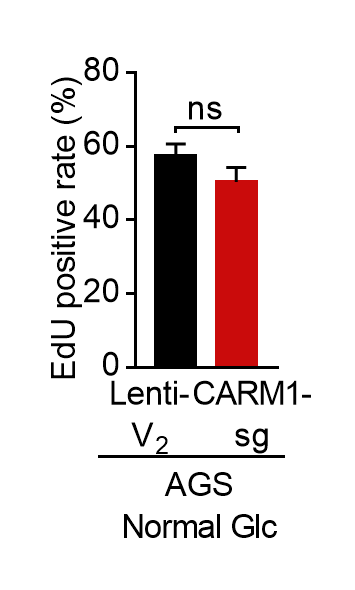

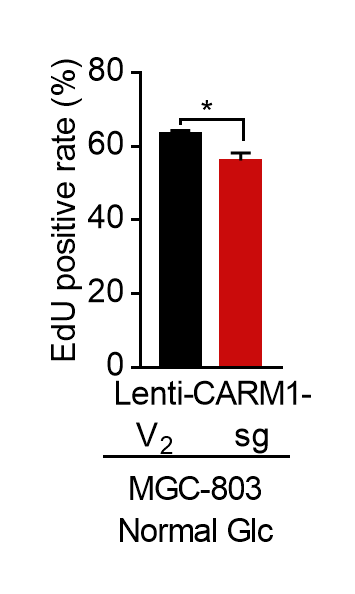

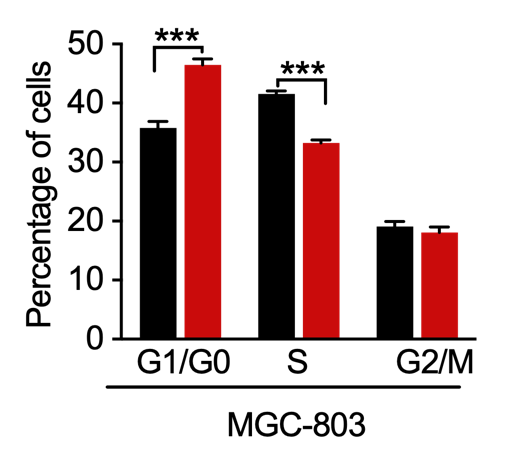

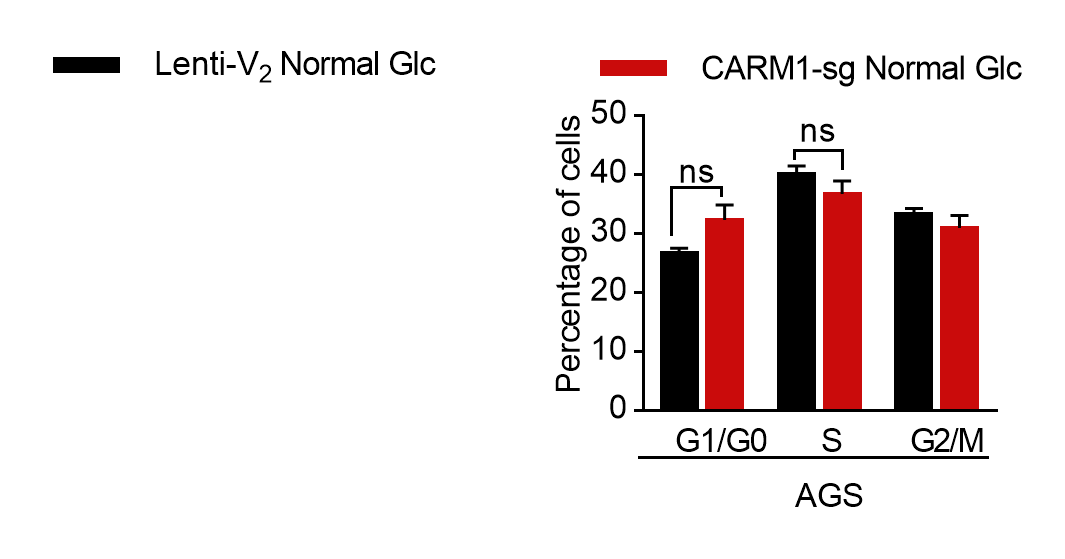

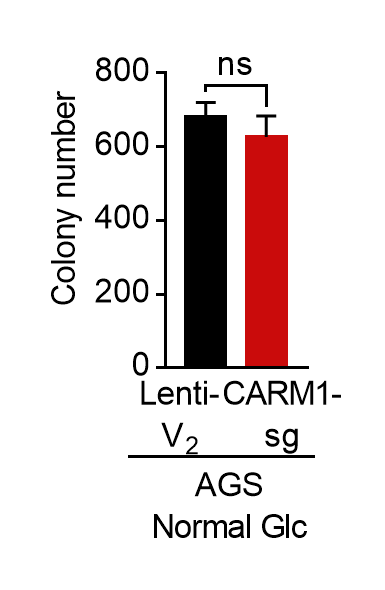

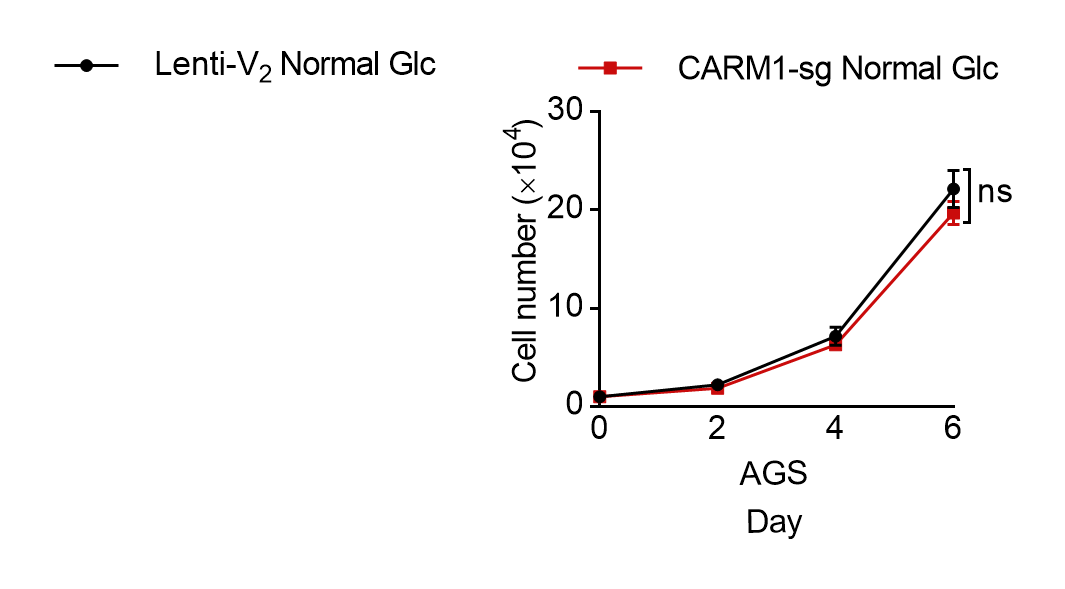

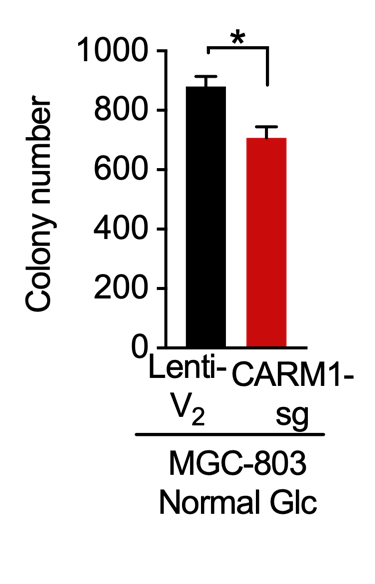


A

B

C

D

E

**Supplementary Figure 3.** **Under normal glucose conditions, *CARM1* knockdown has a less pronounced impact on the malignant behavior of gastric cancer cells compared to low glucose conditions.** A. The proliferation of MGC-803 and AGS cells with parental *CARM1* (Lenti-V_2_) or stable *CARM1* knockdown (CARM1-sg) was evaluated under normal glucose treatment (Normal Glc) by cell counting. B, C. Colony formation assays were performed for MGC-803 and AGS cells with Lenti-V_2_ or CARM1-sg under normal glucose conditions. D. MGC-803 and AGS cells with Lenti-V_2_ or CARM1-sg were cultured under normal glucose conditions, then harvested, fixed, and stained with propidium iodide for DNA content analysis using flow cytometry to determine the percentages of cells in the G1, S, and G2/M phases. E. MGC-803 and AGS cells with Lenti-V_2_ or CARM1-sg were cultured in 6-well plates and exposed to normal glucose for 18 h, followed by treatment with EdU for 2 h and staining with the Apollo reaction cocktail for 30 min to visualize EdU-labeled replicating cells using fluorescence microscopy. All data are shown as mean±SEM. The P values were determined by two-tailed t-tests, and values of P<0.05 were considered statistically significant. ns, not significant; *, P<0.05; ***; P<0.001.

**Supplementary Figure 4**


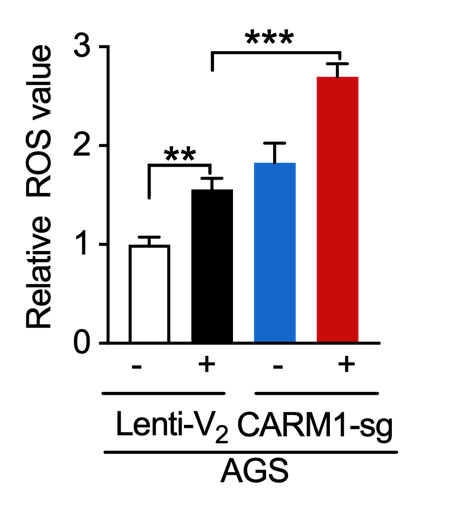

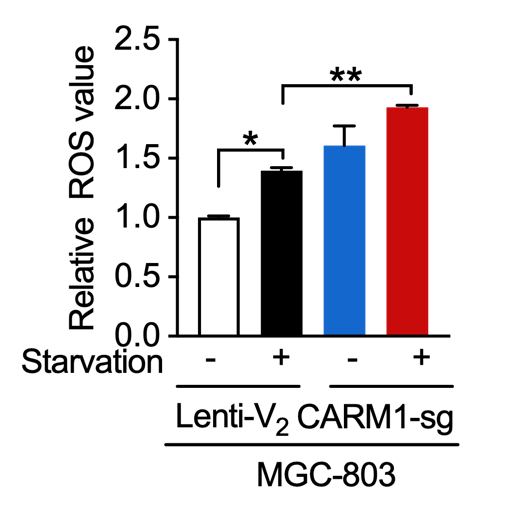


C

B


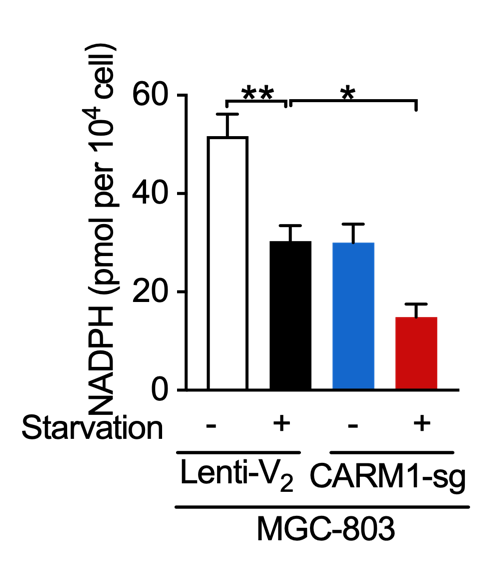

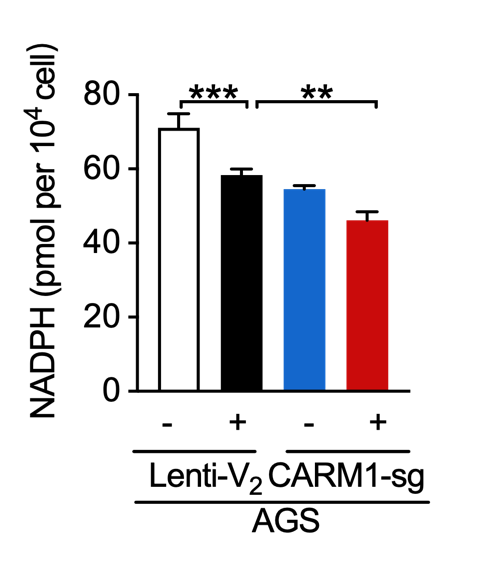

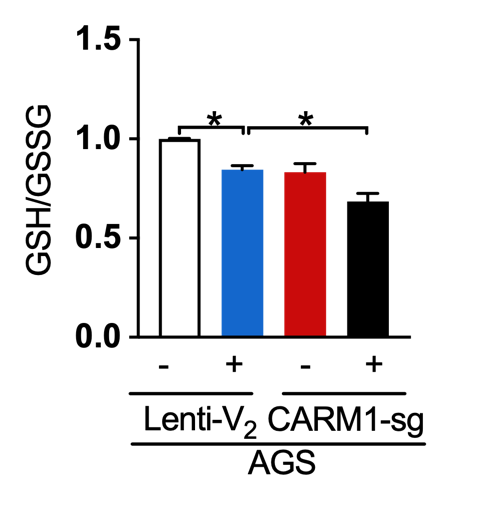

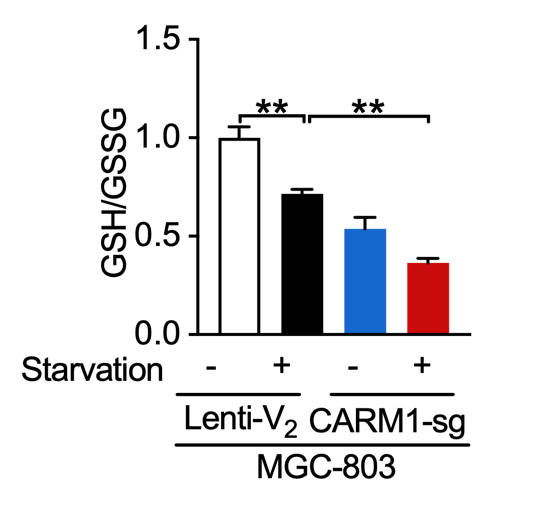


A

E


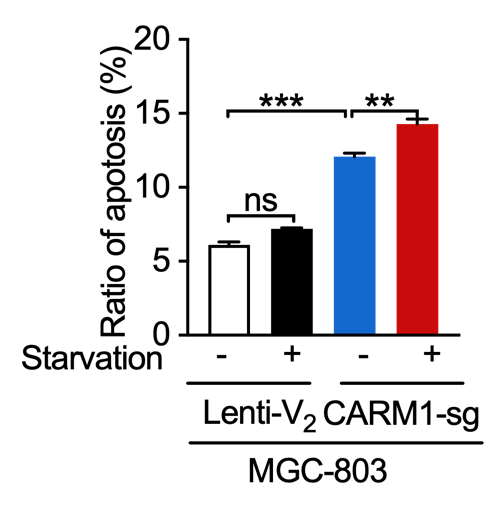

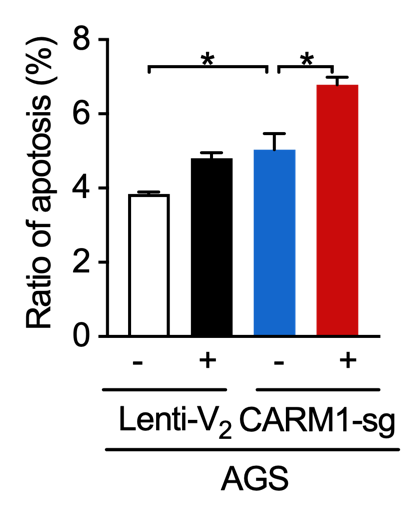


D


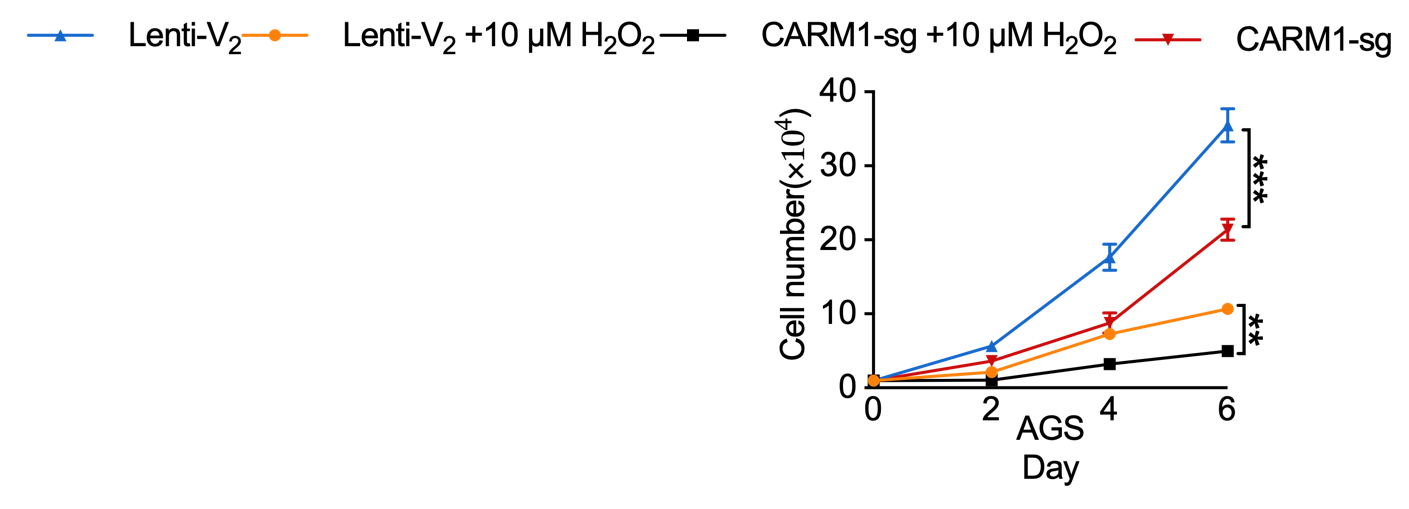

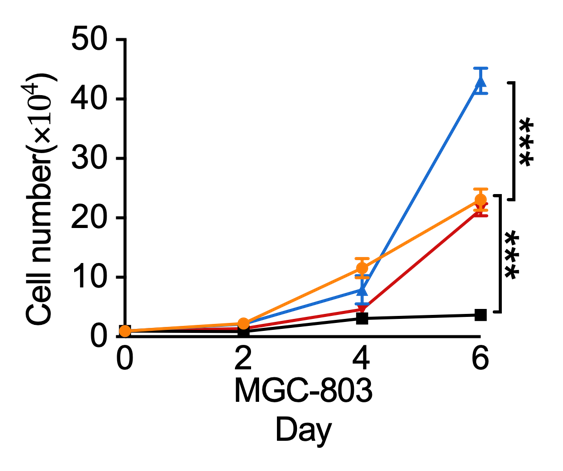


**Supplementary Figure 4. CARM1 inhibits apoptosis induced by glucose starvation.** A. MGC-803 and AGS cells with parental *CARM1* (Lenti-V_2_) or stable *CARM1* knockdown (CARM1-sg) were deprived of glucose or not (as indicated) for measurement of ROS. B, C. The GSH/GSSG ratios (B) and levels of NADPH (C) were measured in MGC-803 and AGS stable cells with Lenti-V_2_ or CARM1-sg under low glucose conditions. D. Apoptosis was measured by Annexin-V/propidium iodide assays for MGC-803 and AGS cells with or without *CARM1* knockdown under low glucose conditions as indicated. Percentage of cells with apoptosis normalized against total cell number. E. Cell proliferation as determined by counts of MGC-803 and AGS cells with Lenti-V_2_ or CARM1-sg treated with or without 10 μmol/L of H_2_O_2_.

**Supplementary Figure 5**


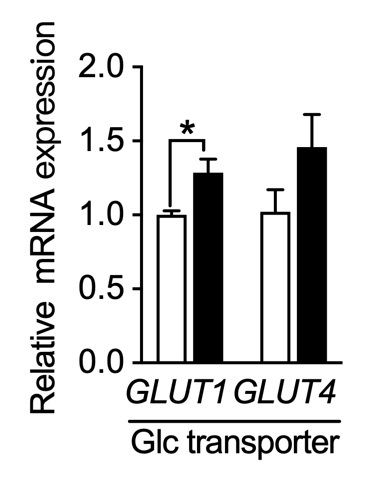

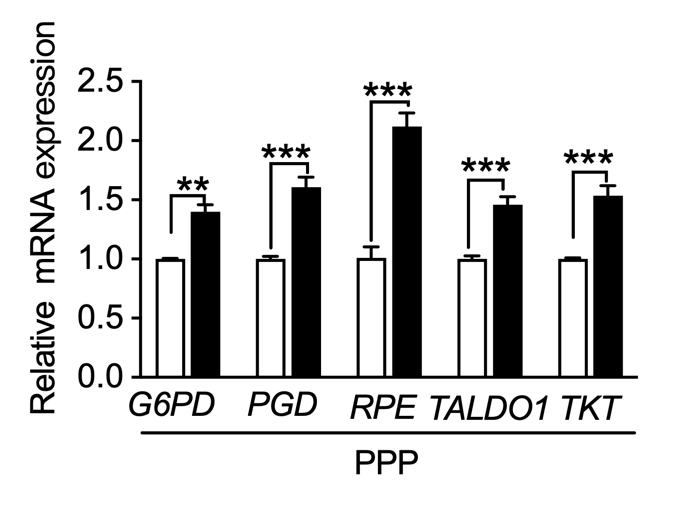

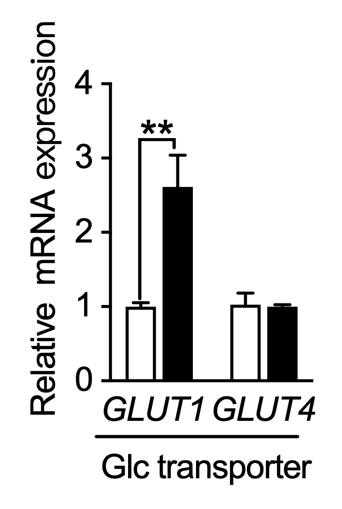

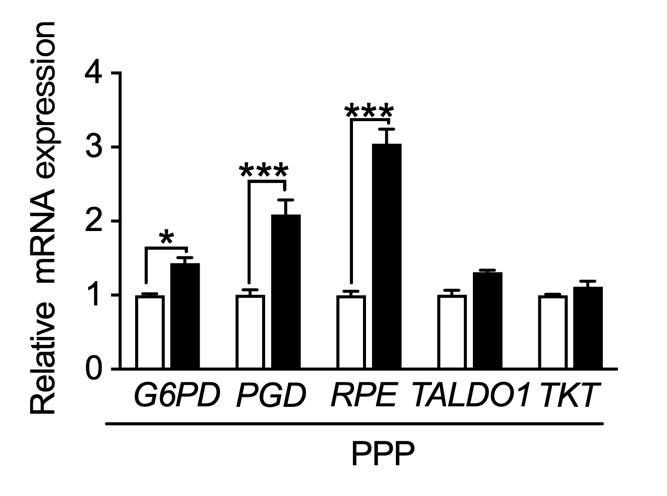


A


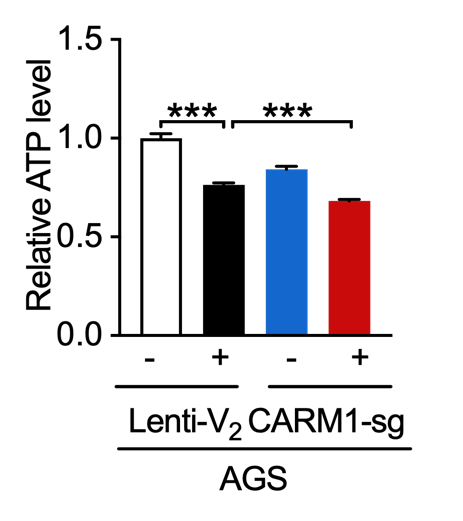

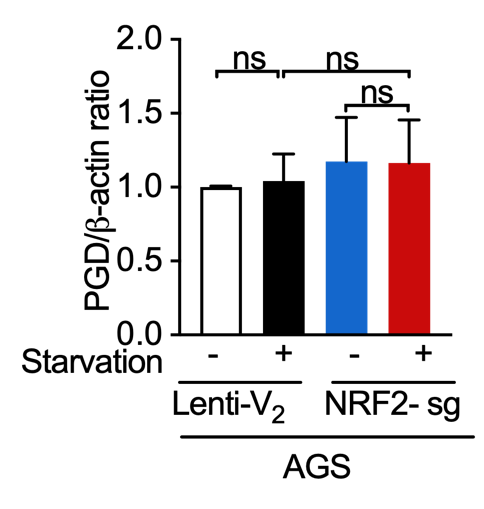

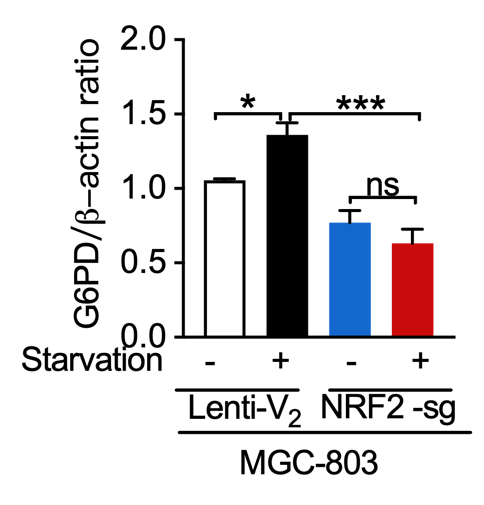

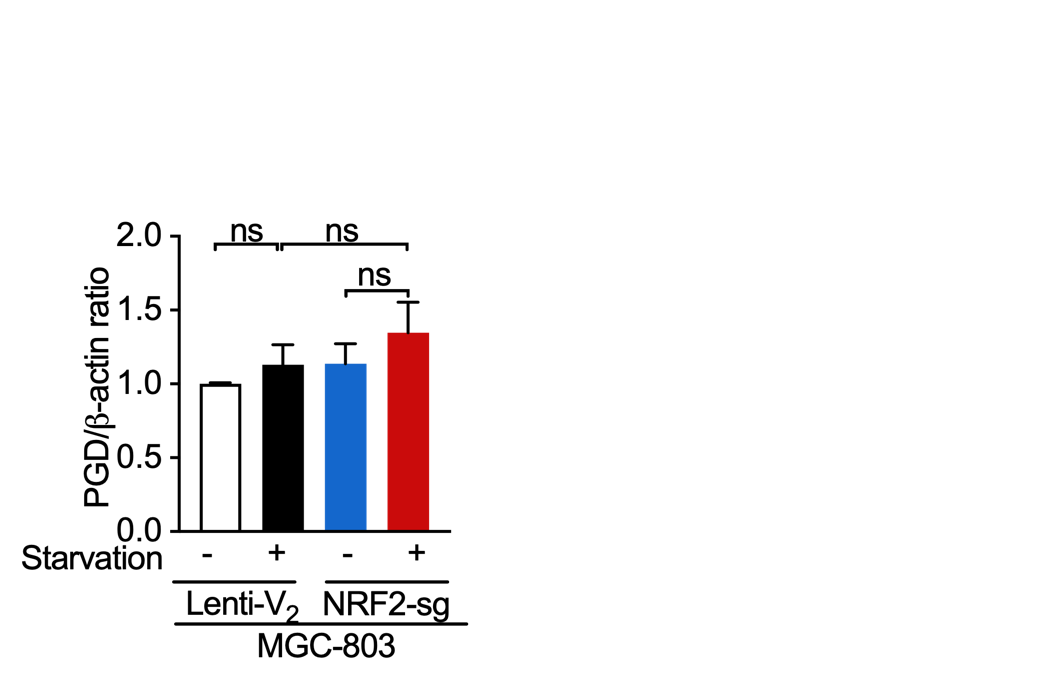

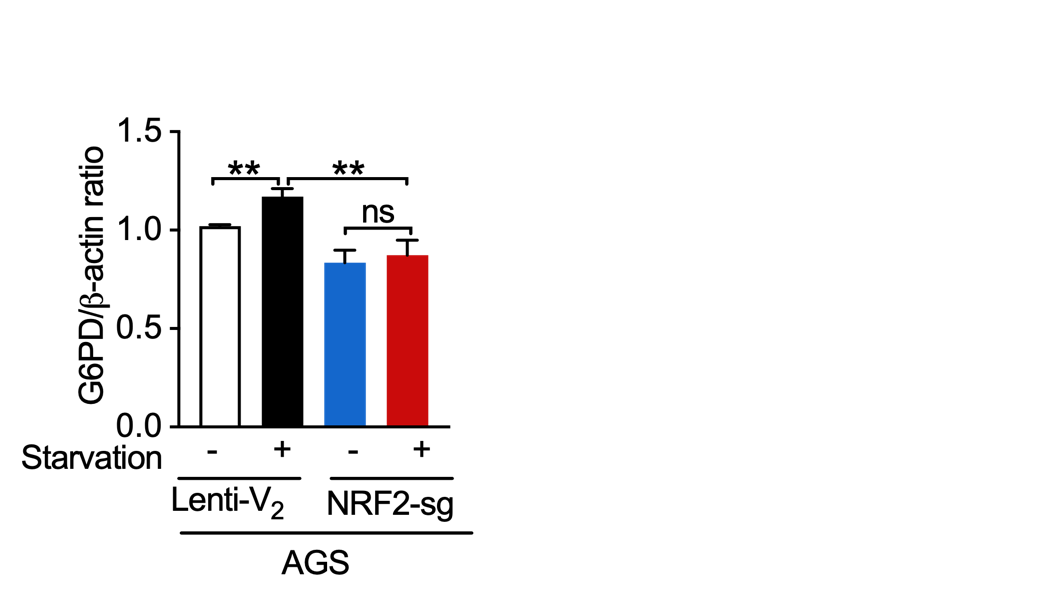


B

C

D


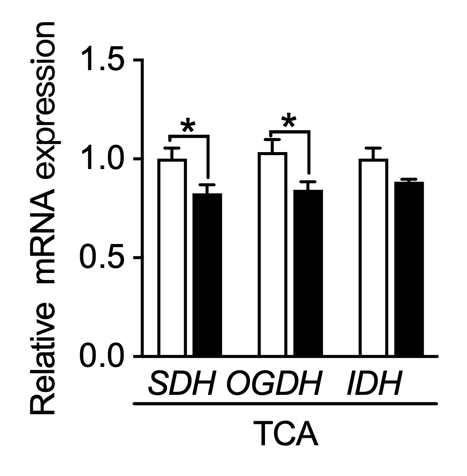

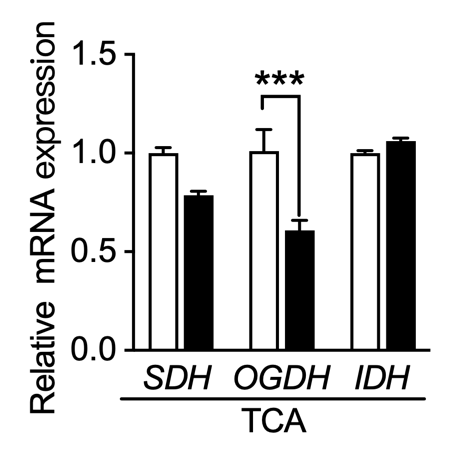

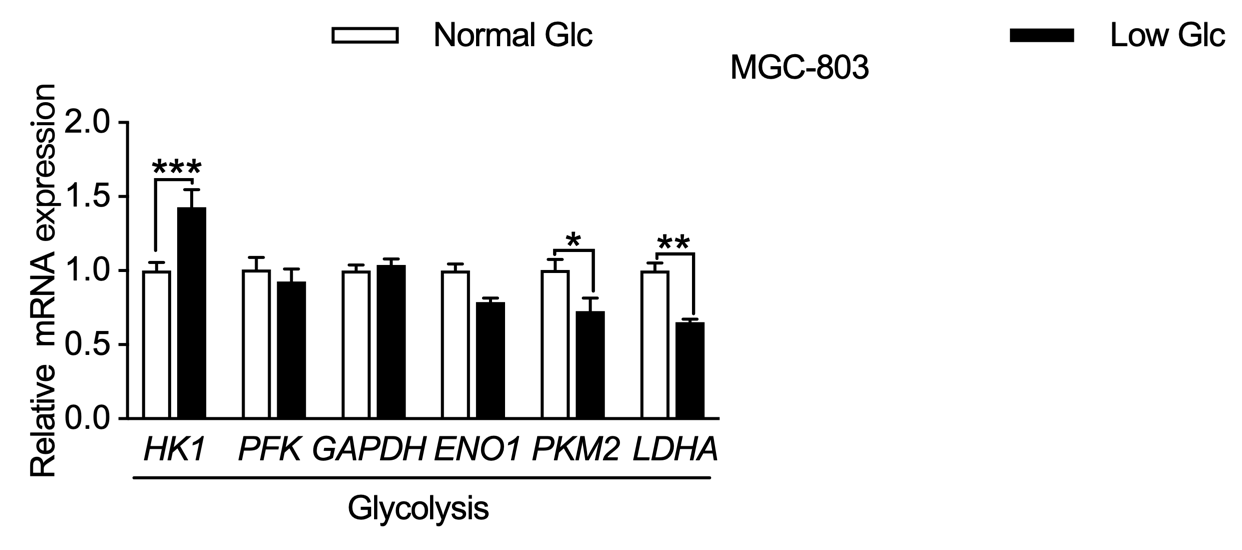

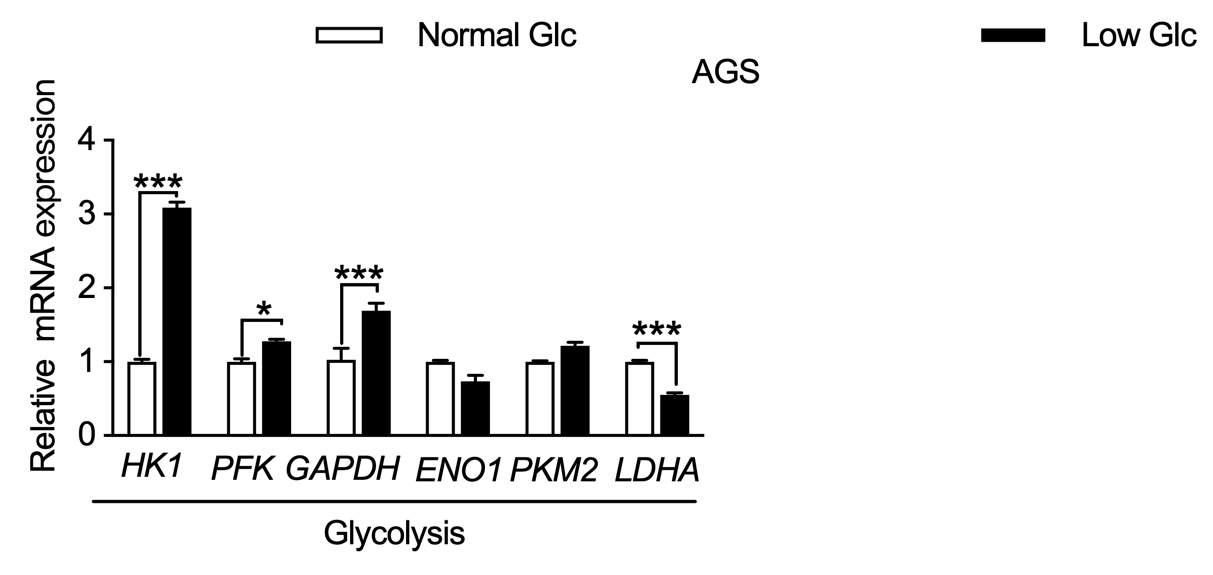


F


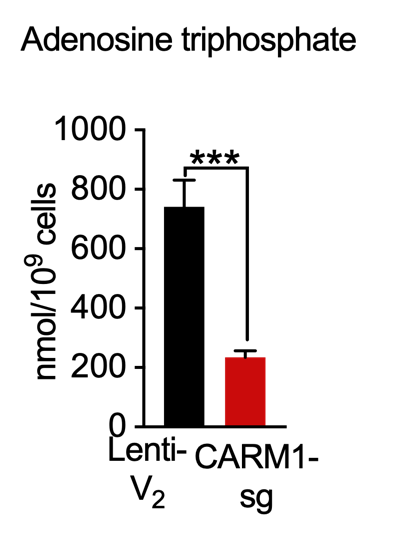


E

MGC-803

AGS


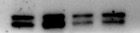

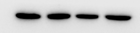

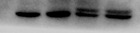


Lenti-V_2_

Glc (mM)

25

25

NRF2

G6PD

β-actin

2.5

NRF2-sg

2.5

PGD

59 kDa

45 kDa

52 kDa


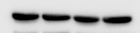


100 kDa

Lenti-V_2_

12

2.5

NRF2-sg

2.5

12


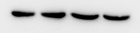

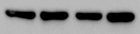

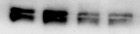

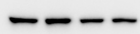

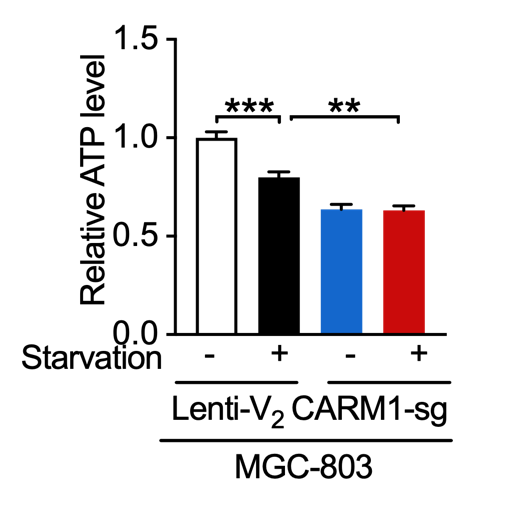


**Supplementary Figure 5. Glucose deprivation induces an increase in the expression of metabolic enzymes involved in the PPP.** A, B. qPCR results of glucose metabolism–related genes in MGC-803 and AGS cells under low glucose (Glc) conditions. C, D. Intracellular ATP levels (measured using HPIC-MS/MS and ATP assay kits) in MGC-803 and AGS cells with parental *CARM1* (Lenti-V_2_) or stable *CARM1* knockdown (CARM1-sg) incubated under glucose starvation conditions or not. E, F. Western blot analysis was performed on MGC-803 and AGS cells with control (Lenti-V_2_) or *NRF2* knockdown (NRF2-sg) treated with low glucose for 18 h (E). Protein expression of G6PD and PGD observed using ImageJ software (F). Data are shown as mean±SEM with multiple replications. ns, not significant; *, P < 0.05; **, P < 0.01; ***, P < 0.001.

**Supplementary Figure 6**


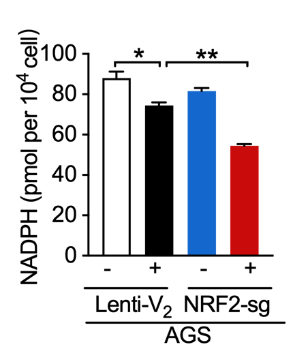

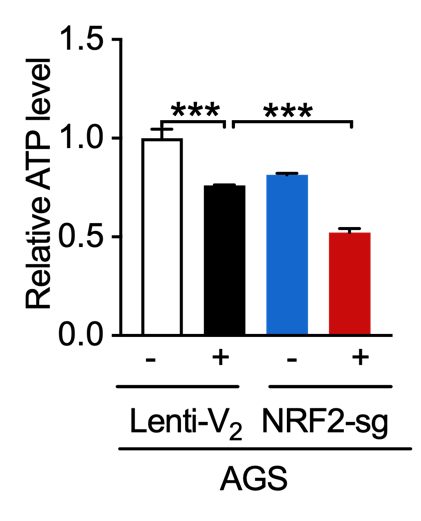

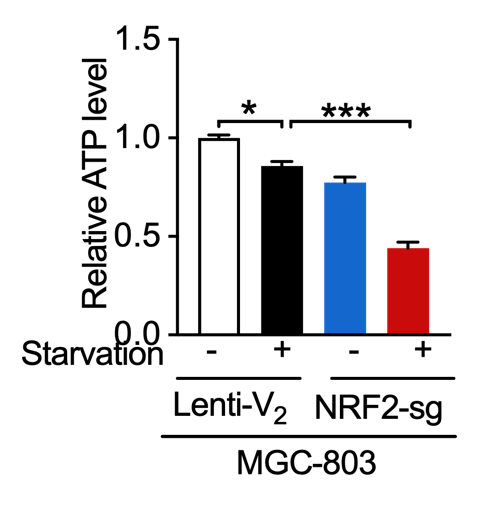

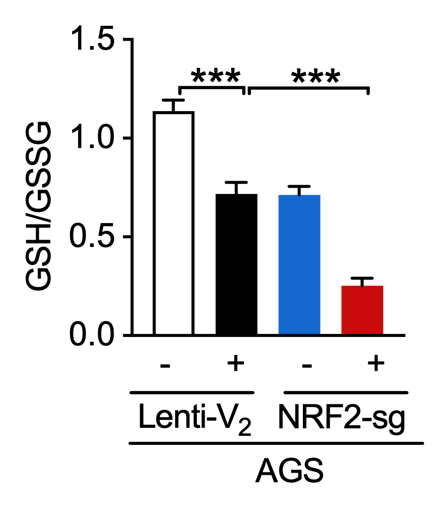

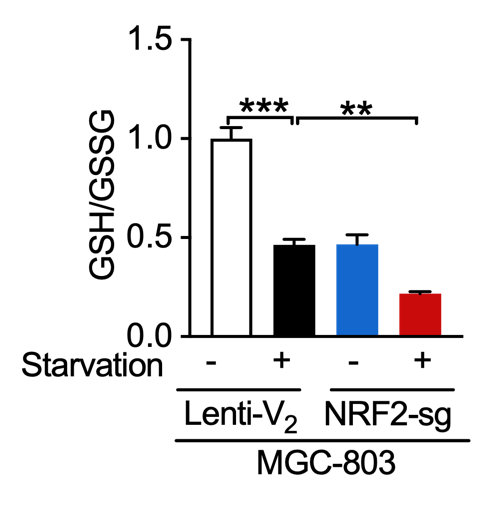

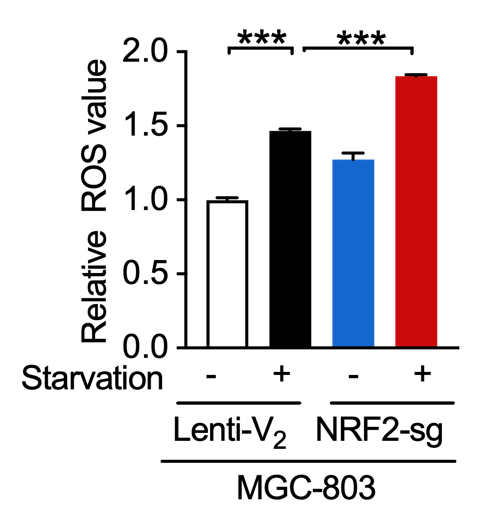

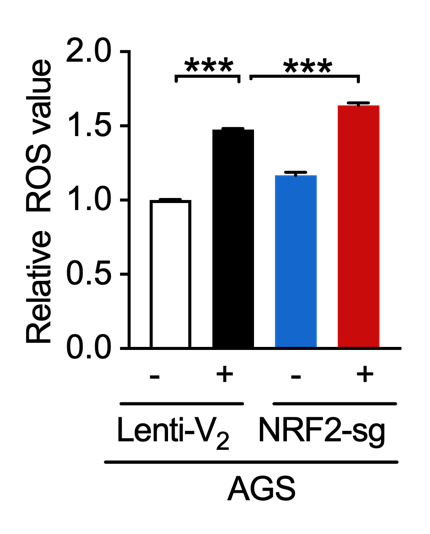


A

B

C

D


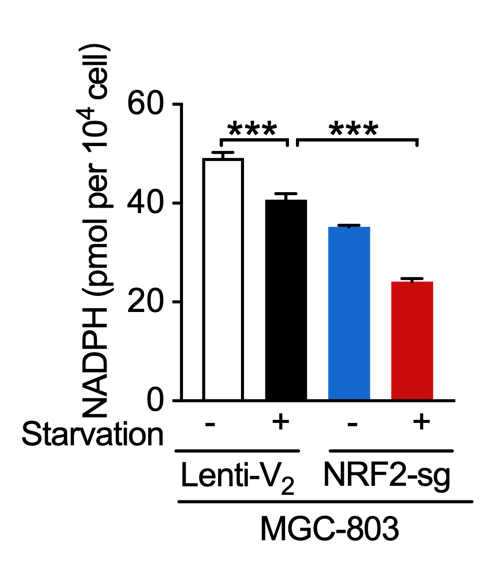


**Supplementary Figure 6. Effects of *NRF2* knockdown on ROS, GSH/GSSG ratio, NADPH content, and intracellular ATP level in MGC-803 and AGS cells.**

A. MGC-803 and AGS cells with parental *NRF2* (Lenti-V_2_) or stable *NRF2* knockdown (NRF2-sg) were deprived of glucose or not (as indicated) for measurement of ROS. B, C. The GSH/GSSG ratios (B) and levels of NADPH (C) were measured in MGC-803 and AGS stable cells with Lenti-V_2_ or CARM1-sg under low glucose conditions. D. Intracellular ATP levels (measured using ATP assay kits) in MGC-803 and AGS cells with parental *NRF2* (Lenti-V_2_) or stable *NRF2* knockdown (NRF2-sg) incubated under glucose starvation conditions or not. Data are shown as mean±SEM with multiple replications. *, P < 0.05; **, P < 0.01; ***, P < 0.001.

**Supplementary Figure 7**


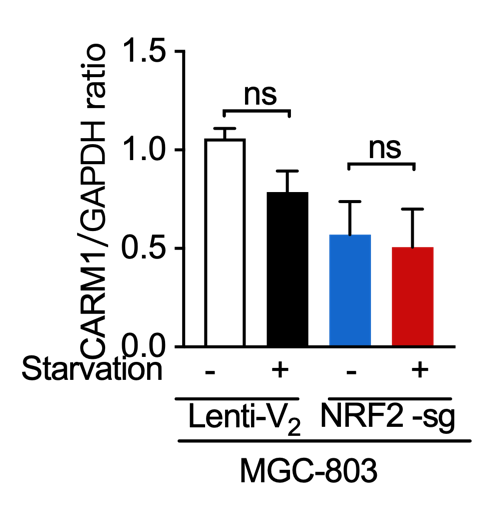

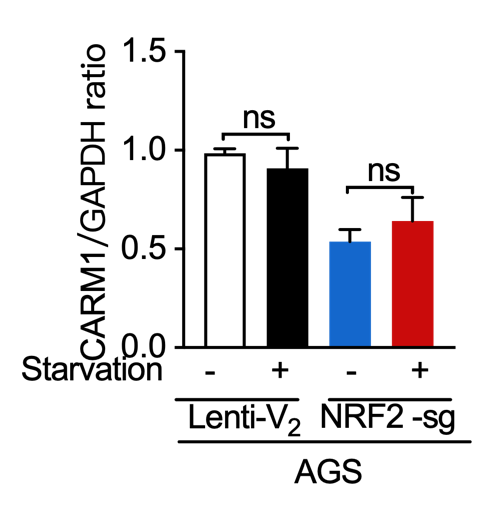

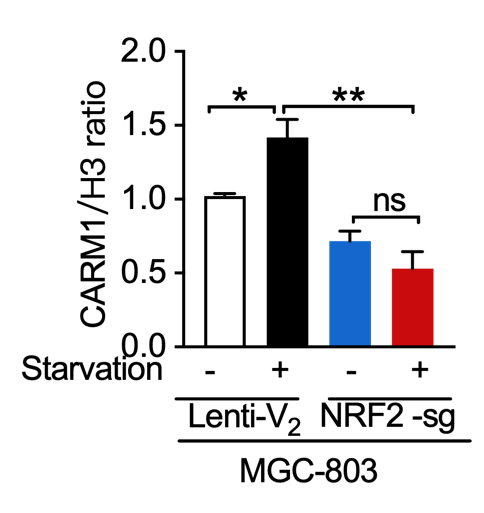

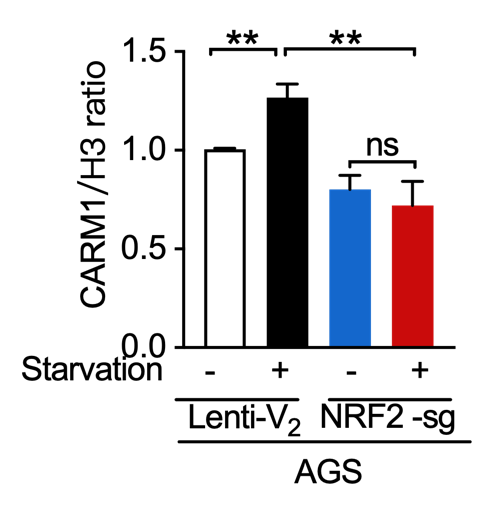


B

C


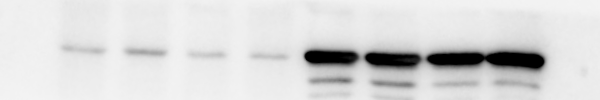

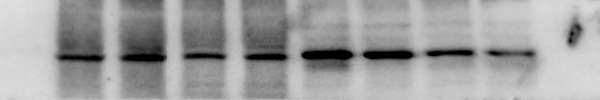

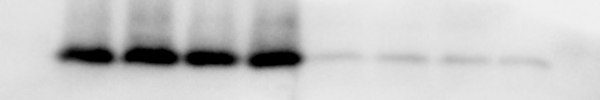


CARM1

H3

GAPDH

12

2.5

12

2.5

12

2.5

12

2.5

CARM1-sg

Lenti-V_2_

CARM1-sg

Lenti-V_2_

Glc (mM)

Nucleus

Cytoplasm

AGS


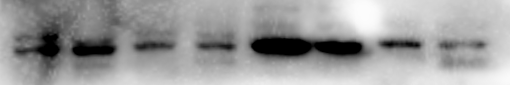

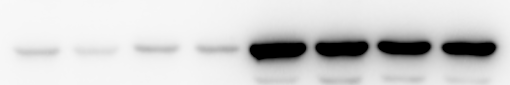

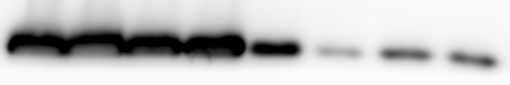


CARM1

H3

GAPDH

CARM1-sg

Lenti-V_2_

Glc (mM)

25

2.5

25

2.5

CARM1-sg

Lenti-V_2_

25

2.5

25

2.5

Nucleus

Cytoplasm

A

MGC-803

**Supplementary Figure 7.** **CARM1 protein levels in the nucleus are increased after glucose starvation.** A, B.  MGC-803 and AGS cells with parental CARM1 (Lenti-V_2_) or stable CARM1 knockdown (CARM1-sg) were treated with various concentrations of glucose (Glc) for 18 h as indicated for nuclear isolation, followed by Western blotting for CARM1, GAPDH, and Histone H3. Results are representative of three independent experiments. Data are shown as mean±SEM with multiple replications. ns, not significant; *, P<0.05, **, P < 0.01.

**Supplementary Figure 8**

A


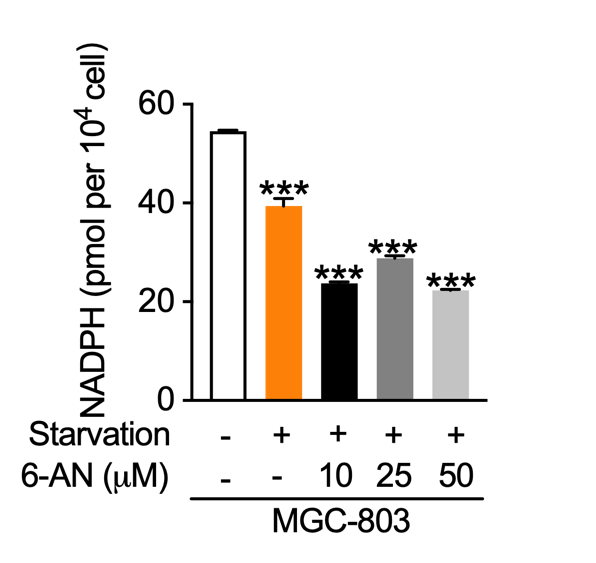

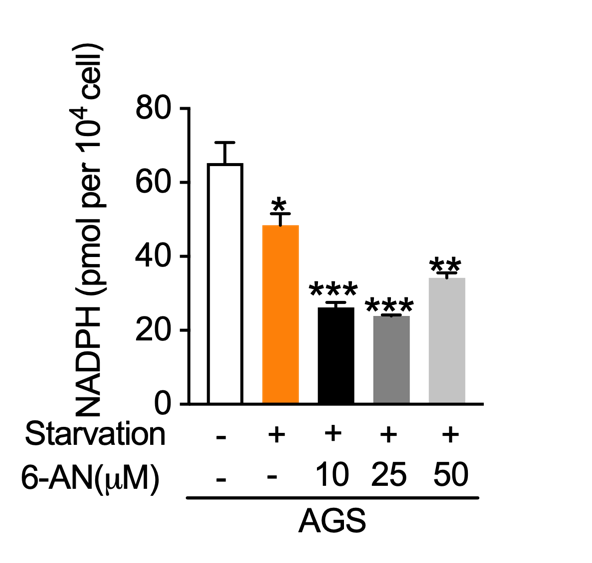


B


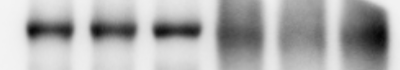

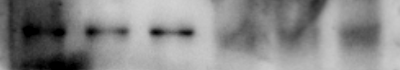

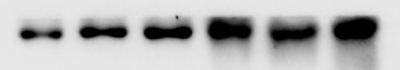


G6PD

CARM1

β-actin

Lenti-V_2_

CARM1-sg

Mouse NO.

1 2 3

1 2 3

62 kDa

59 kDa

45 kDa


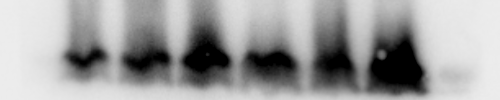

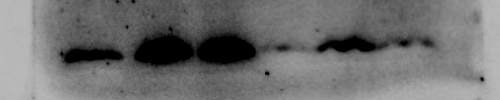


H3R17me

H3

17 kDa

17 kDa

**Supplementary Figure 8. 6-AN inhibits NADPH production and cell growth by suppressing the PPP.** A. MGC-803 and AGS cells were treated with different concentrations of glucose and supplemented or not with the indicated concentration of 6-AN for measurement of NADPH. Results are representative of three independent experiments. Data are shown as mean±SEM with multiple replications. *, P<0.05, **, P<0.01, and ***, P<0.001. B. Protein expression of G6PD, CARM1, and H3R17me2 in collected tumors as determined by Western blot analysis.
