# Supplementary material for: The NRF2-CARM1 axis links glucose sensing to transcriptional and epigenetic regulation of the pentose phosphate pathway in gastric cancer
Source: Cell Death Dis. 2024 Sep 12;15(9):670. doi: 10.1038/s41419-024-07052-3 (PMC11393079; doi:10.1038/s41419-024-07052-3)
Supplement: Supplementary file 2 — Supplementary table 1 [file 41419_2024_7052_MOESM2_ESM.docx]

Supplementary Table 1. RT-qPCR primer sequences.

| Gene name | Sequence 5'-3' | Specificity | Note |
| --- | --- | --- | --- |
| *ACTB-F* | TCATGAAGTGTGACGTGGACATC | Human | RT-PCR |
| *ACTB-R* | CAGGAGGAGCAATGATCTTGATCT | Human |  |
| *GLUT1-F* | CAGGAGATGAAGGAAGAG | Human |  |
| *GLUT1-R* | TCGTGGAGTAATAGAAGAC | Human |  |
| *GLUT4-F* | CCATCCTGATGACTGTGGCTCT | Human |  |
| *GLUT4-R* | GCCACGATGAACCAAGGAATGG | Human |  |
| *HK2-F* | GAGTTTGACCTGGATGTGGTTGC | Human |  |
| *HK2-R* | CCTCCATGTAGCAGGCATTGCT | Human |  |
| *PFK1-F* | GCTTCTAGCTCATGTCAGACCC | Human |  |
| *PFK1-R* | CCAATCCTCACAGTGGAGCGAA | Human |  |
| *GAPDH-F* | GTCTCCTCTGACTTCAACAGCG | Human |  |
| *GAPDH-R* | ACCACCCTGTTGCTGTAGCCAA | Human |  |
| *ENO1-F* | AGTCAACCAGATTGGCTCCGTG | Human |  |
| *ENO1-R* | CACAACCAGGTCAGCGATGAAG | Human |  |
| *PKM2-F* | ATGGCTGACACATTCCTGGAGC | Human |  |
| *PKM2-R* | CCTTCAACGTCTCCACTGATCG | Human |  |
| *LDHA-F* | GGATCTCCAACATGGCAGCCTT | Human |  |
| *LDHA-R* | AGACGGCTTTCTCCCTCTTGCT | Human |  |
| *G6PD-F* | TGACCTGGCCAAGAAGAAGA | Human |  |
| *G6PD-R* | CAAAGAAGTCCTCCAGCTTG | Human |  |
| *PGD-F* | ATATAGGGACACCACAAGACGG | Human |  |
| *PGD-R* | GCATGAGCGATGGGCCATA | Human |  |
| *RPE-F* | CGTAATGGACGGACATGCAC | Human |  |
| *RPE-R* | ATGGCAAGGCCAACCTTCAT | Human |  |
| *TALDO1-F* | GTCATCAACCTGGGAAGGAA | Human |  |
| *TALDO1-R* | CAACAAATGGGGAGATGAGG | Human |  |
| *TKT-F* | GCTGAACCTGAGGAAGATCA | Human |  |
| *TKT-R* | TGTCGAAGTATTTGCCGGTG | Human |  |
| *SOD1-F* | CTCACTCTCAGGAGACCATTGC | Human |  |
| *SOD1-R* | CCACAAGCCAAACGACTTCCAG | Human |  |
| *NQO1-F* | CCTGCCATTCTGAAAGGCTGGT | Human |  |
| *NQO1-R* | GTGGTGATGGAAAGCACTGCCT | Human |  |
| *GCLC-F* | GGAAGTGGATGTGGACACCAGA | Human |  |
| *GCLC-R* | GCTTGTAGTCAGGATGGTTTGCG | Human |  |
| *SDH-F* | CTGGAGGCTGAGTGCCGTTT | Human |  |
| *SDH-R* | TCTGGGATAGGTCGGTCCTGAA | Human |  |
| *OGDH-F* | GAGGCTGTCATGTGTGCA | Human |  |
| *OGDH-R* | TACATGAGCGGCTGCGTGAACA | Human |  |
| *IDH-F* | CTCTGTGGCCCAAGGGTATG | Human |  |
| *IDH-R* | GGATTGGTGGACGTCTCCTG | Human |  |
