# Supplementary material for: The NRF2-CARM1 axis links glucose sensing to transcriptional and epigenetic regulation of the pentose phosphate pathway in gastric cancer
Source: Cell Death Dis. 2024 Sep 12;15(9):670. doi: 10.1038/s41419-024-07052-3 (PMC11393079; doi:10.1038/s41419-024-07052-3)
Supplement: Supplementary file 3 — Supplementary table 2 [file 41419_2024_7052_MOESM3_ESM.docx]

Supplementary Table 2. ChIP-qPCR primer sequences.

| Primer name | Sequence 5'-3' | Specificity | Product length (bp) |
| --- | --- | --- | --- |
| G6PD region 1 F | TCAGGAGTTCAAGACCAGACT | human | 177 |
| G6PD region 1 R | AGTGGCATGATCTCAGCTCA | human |  |
| G6PD region 2F | TGACCTCCGTGCTATTCCTC | human | 196 |
| G6PD region 2R | ACCAAACTTGACTGCGCTCT | human |  |
| G6PD region 3F | CCAAGTAACCTGGGTCCTGA | human | 178 |
| G6PD region 3R | GCTGACAGGTGTGGTCCTTT | human |  |
| PGD region 1F | TTGGGGTGATGGAAGTTTTC | human | 215 |
| PGD region 1R | ACTACAGGCATGCAACACCA | human |  |
| PGD region 2F | GCGAGACTCCGTCTCAAAAA | human | 223 |
| PGD region 2R | CGGTATTGAACCCCTTCTCA | human |  |
| PGD region 3F | GGTCCTGCGTGAGTTGCTAT | human | 198 |
| PGD region 3R | TTAGACCATCCGAGGCAAAC | human |  |
