# Supplementary material for: The NRF2-CARM1 axis links glucose sensing to transcriptional and epigenetic regulation of the pentose phosphate pathway in gastric cancer
Source: Cell Death Dis. 2024 Sep 12;15(9):670. doi: 10.1038/s41419-024-07052-3 (PMC11393079; doi:10.1038/s41419-024-07052-3)
Supplement: Supplementary file 4 — full length uncropped original western blots [file 41419_2024_7052_MOESM4_ESM.pptx]

## Slide 1
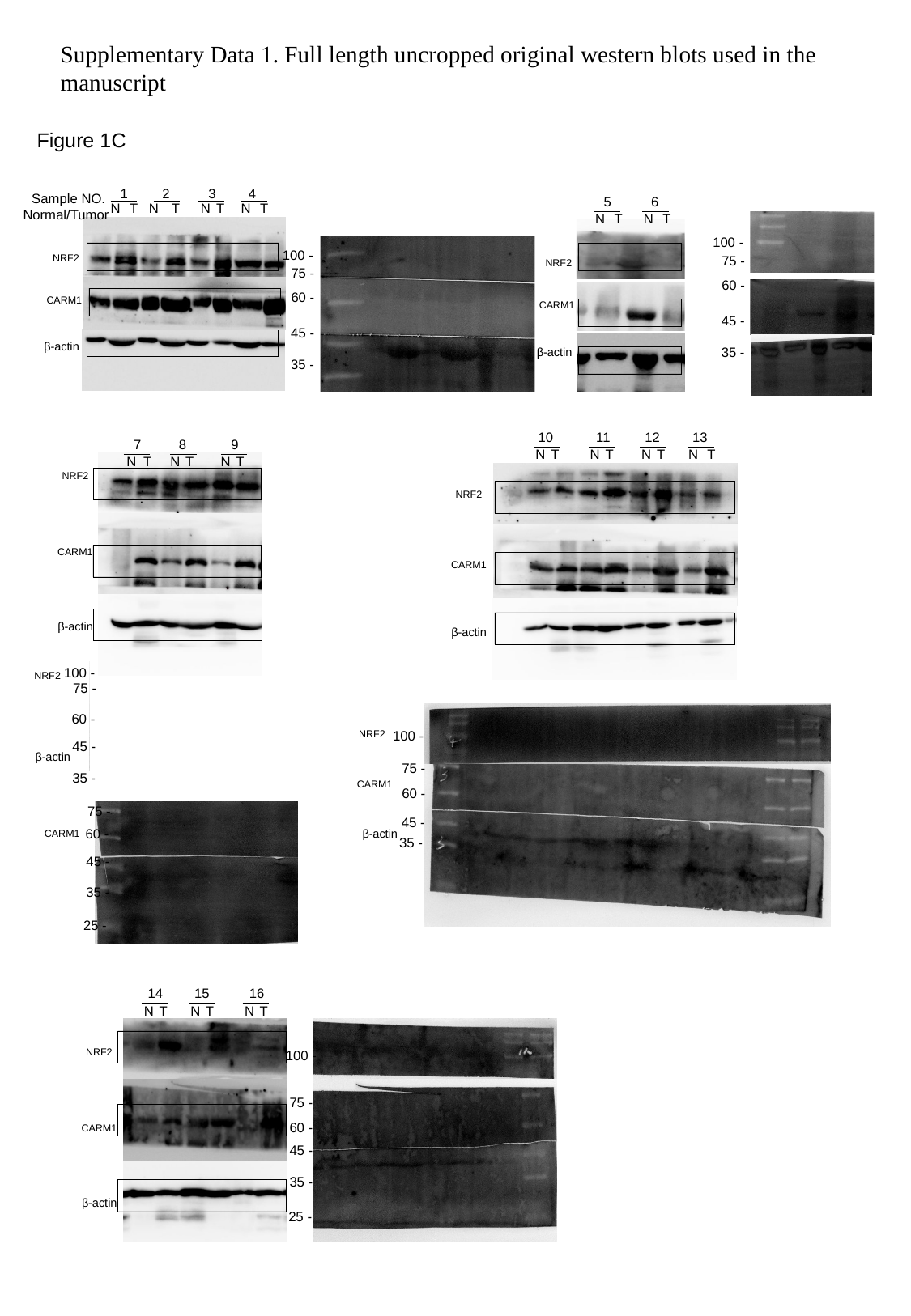

Supplementary Data 1. Full length uncropped original western blots used in the manuscript
Figure 1C
1
2
3
4
Sample NO.
5
6
N
T
N
T
N
T
N
T
Normal/Tumor
N
T
N
T
100 -
100 -
NRF2
75 -
NRF2
75 -
60 -
60 -
CARM1
CARM1
45 -
45 -
β-actin
35 -
β-actin
35 -
10
11
12
13
7
8
9
N
T
N
T
N
T
N
T
N
T
N
T
N
T
NRF2
NRF2
CARM1
CARM1
β-actin
β-actin
100 -
NRF2
75 -
60 -
NRF2
100 -
45 -
β-actin
75 -
35 -
CARM1
60 -
75 -
45 -
60 -
β-actin
CARM1
35 -
45 -
35 -
25 -
14
15
16
N
T
N
T
N
T
NRF2
100 -
75 -
60 -
CARM1
45 -
35 -
β-actin
25 -

## Slide 2
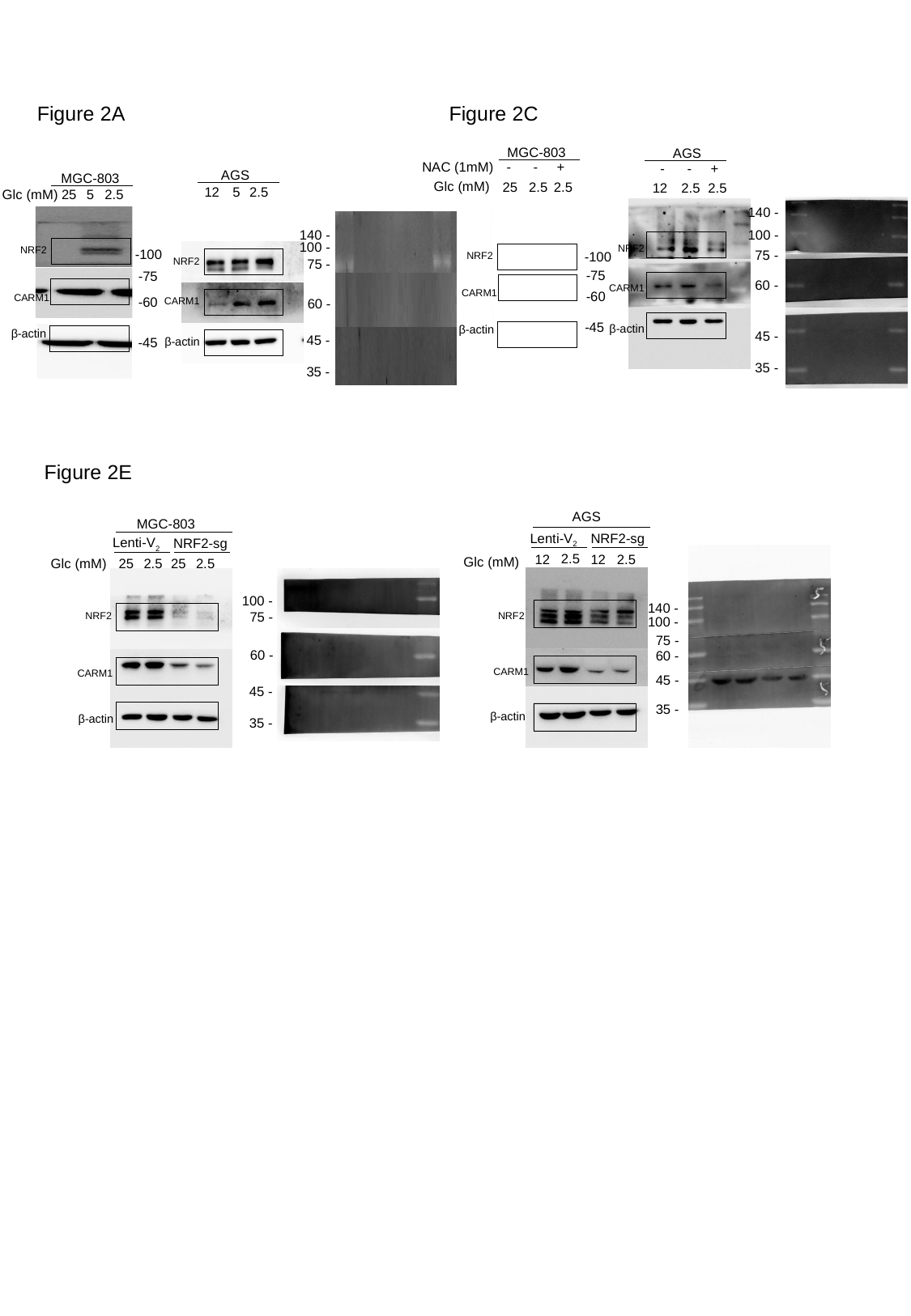

Figure 2A
Figure 2C
MGC-803
AGS
NAC (1mM)
 -
-
 +
 -
-
 +
AGS
MGC-803
Glc (mM)
25
2.5
2.5
2.5
2.5
12
12
5
2.5
Glc (mM)
25
5
2.5
140 -
100 -
NRF2
75 -
60 -
CARM1
β-actin
45 -
35 -
 -100
NRF2
-75
CARM1
-60
-45
β-actin
140 -
100 -
NRF2
 -100
NRF2
75 -
-75
CARM1
-60
CARM1
60 -
β-actin
45 -
β-actin
-45
35 -
Figure 2E
AGS
MGC-803
Lenti-V2
NRF2-sg
Lenti-V2
NRF2-sg
2.5
12
12
2.5
NRF2
CARM1
β-actin
Glc (mM)
Glc (mM)
2.5
25
25
2.5
NRF2
CARM1
β-actin
100 -
140 -
75 -
100 -
75 -
60 -
60 -
45 -
45 -
35 -
35 -

## Slide 3
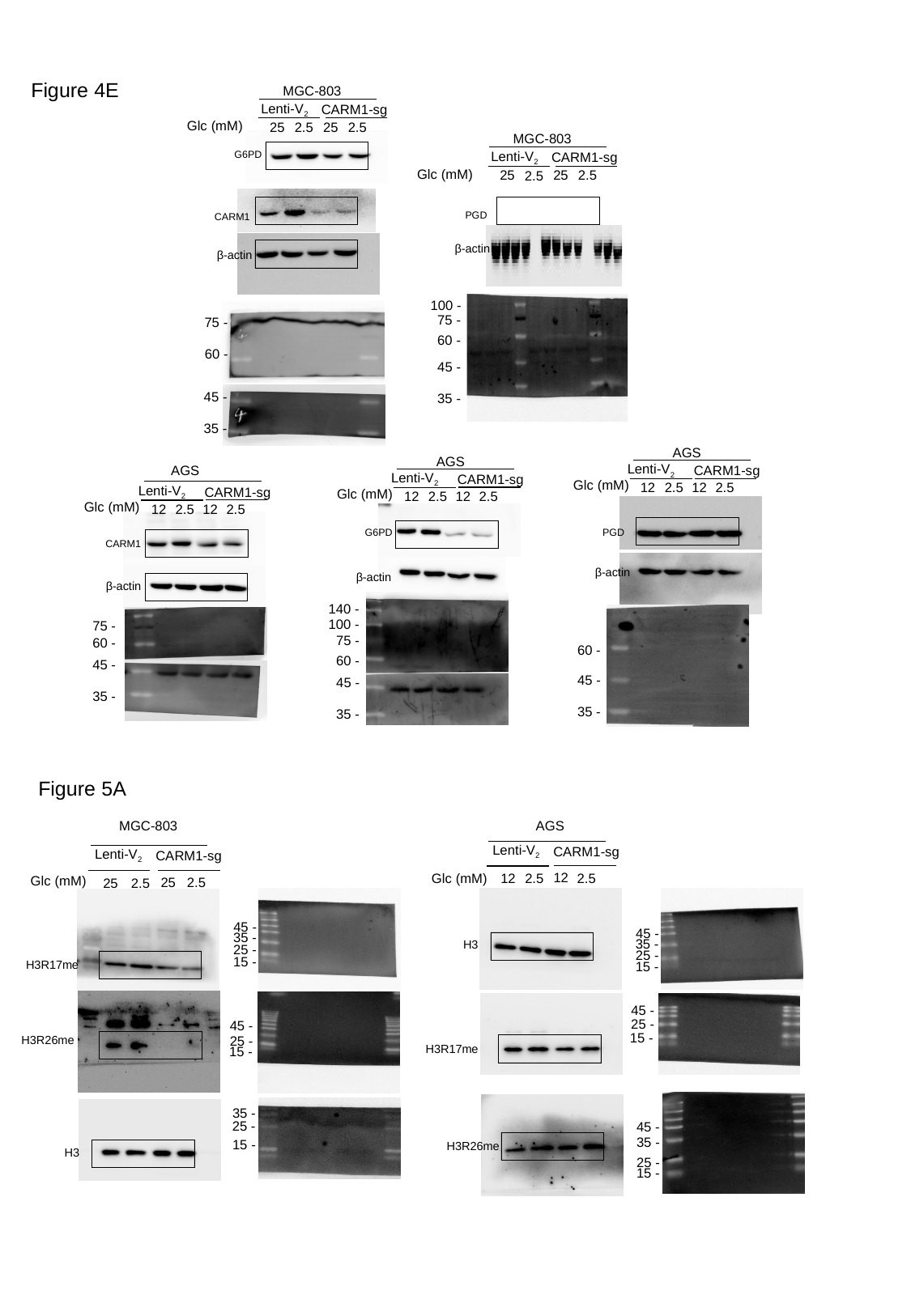

Figure 4E
MGC-803
Lenti-V2
CARM1-sg
Glc (mM)
25
2.5
25
2.5
MGC-803
Lenti-V2
G6PD
CARM1-sg
Glc (mM)
25
2.5
25
2.5
PGD
CARM1
β-actin
β-actin
100 -
75 -
60 -
45 -
35 -
75 -
60 -
45 -
35 -
AGS
AGS
Lenti-V2
AGS
CARM1-sg
Lenti-V2
CARM1-sg
Glc (mM)
12
2.5
12
2.5
Lenti-V2
CARM1-sg
Glc (mM)
12
2.5
12
2.5
Glc (mM)
12
2.5
12
2.5
PGD
G6PD
CARM1
β-actin
β-actin
β-actin
140 -
75 -
60 -
45 -
35 -
100 -
75 -
60 -
60 -
45 -
45 -
35 -
35 -
Figure 5A
AGS
MGC-803
Lenti-V2
CARM1-sg
Lenti-V2
CARM1-sg
12
Glc (mM)
2.5
12
2.5
Glc (mM)
25
2.5
25
2.5
45 -
35 -
25 -
15 -
45 -
35 -
H3
25 -
H3R17me
15 -
45 -
25 -
15 -
45 -
25 -
H3R26me
H3R17me
15 -
35 -
25 -
15 -
45 -
35 -
H3R26me
H3
25 -
15 -

## Slide 4
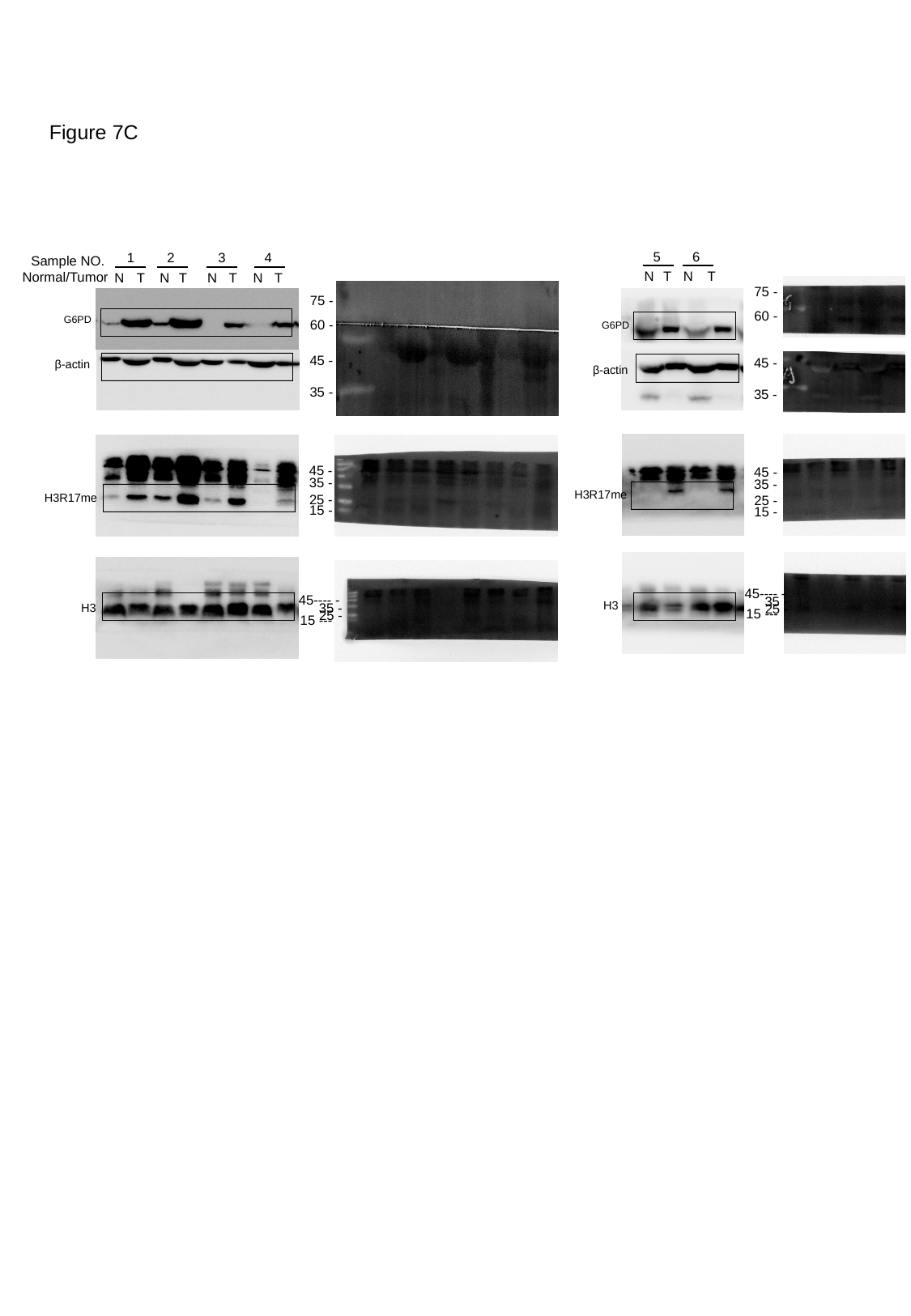

Figure 7C
5
6
1
2
3
4
Sample NO.
N
T
N
T
Normal/Tumor
N
T
N
T
N
T
N
T
75 -
75 -
60 -
G6PD
60 -
G6PD
45 -
45 -
β-actin
β-actin
35 -
35 -
45 -
45 -
35 -
35 -
H3R17me
H3R17me
25 -
25 -
15 -
15 -
45---- -
45---- -
35 -
H3
35 -
H3
25 -
15 ---
25 -
15 ---

## Slide 5
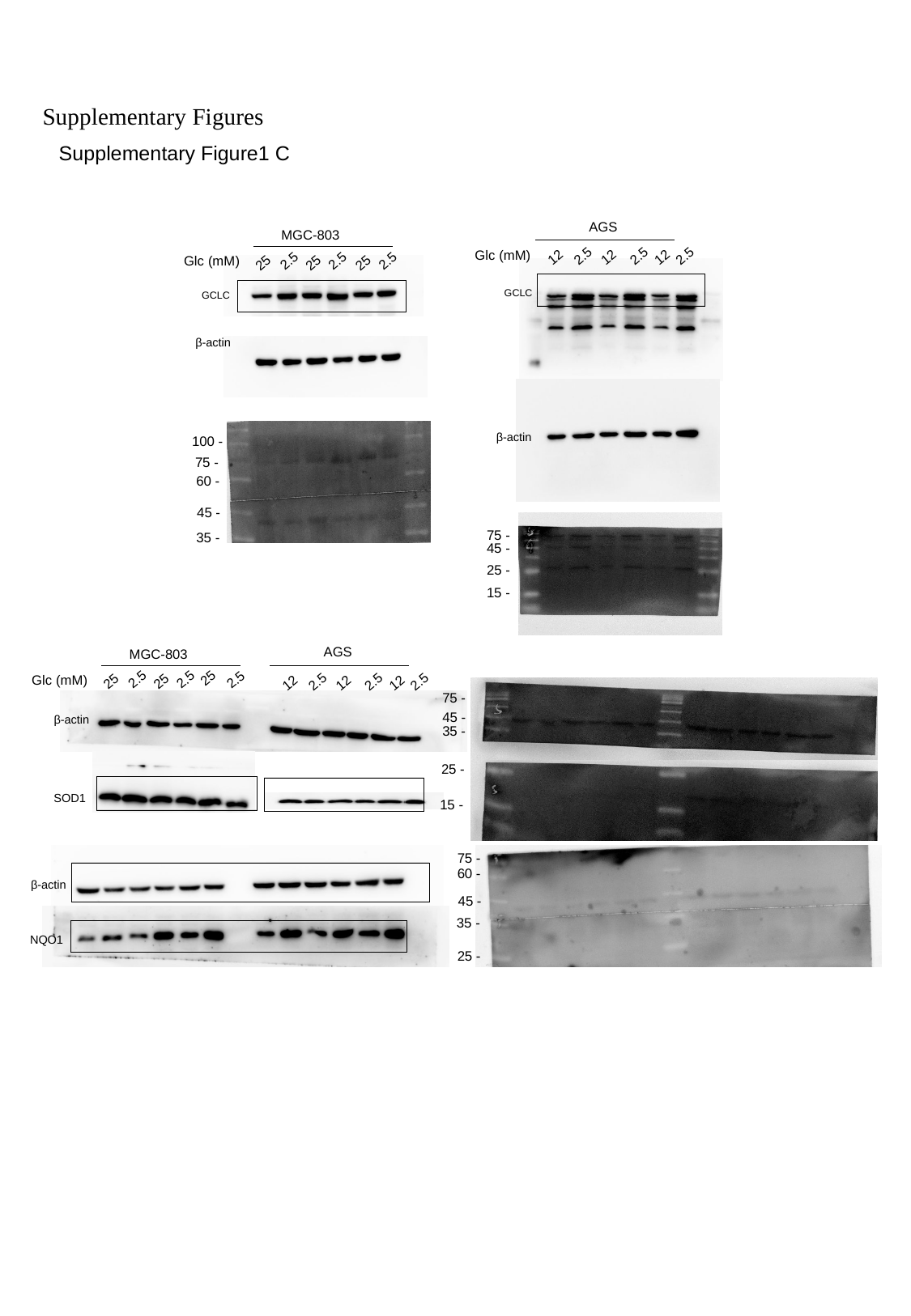

Supplementary Figures
Supplementary Figure1 C
AGS
MGC-803
12
12
12
2.5
2.5
2.5
Glc (mM)
25
2.5
25
2.5
25
2.5
Glc (mM)
GCLC
GCLC
β-actin
β-actin
100 -
75 -
60 -
45 -
75 -
35 -
45 -
25 -
15 -
AGS
MGC-803
25
25
2.5
25
2.5
2.5
12
12
12
2.5
2.5
2.5
Glc (mM)
75 -
45 -
β-actin
35 -
25 -
SOD1
15 -
75 -
60 -
β-actin
45 -
35 -
NQO1
25 -

## Slide 6
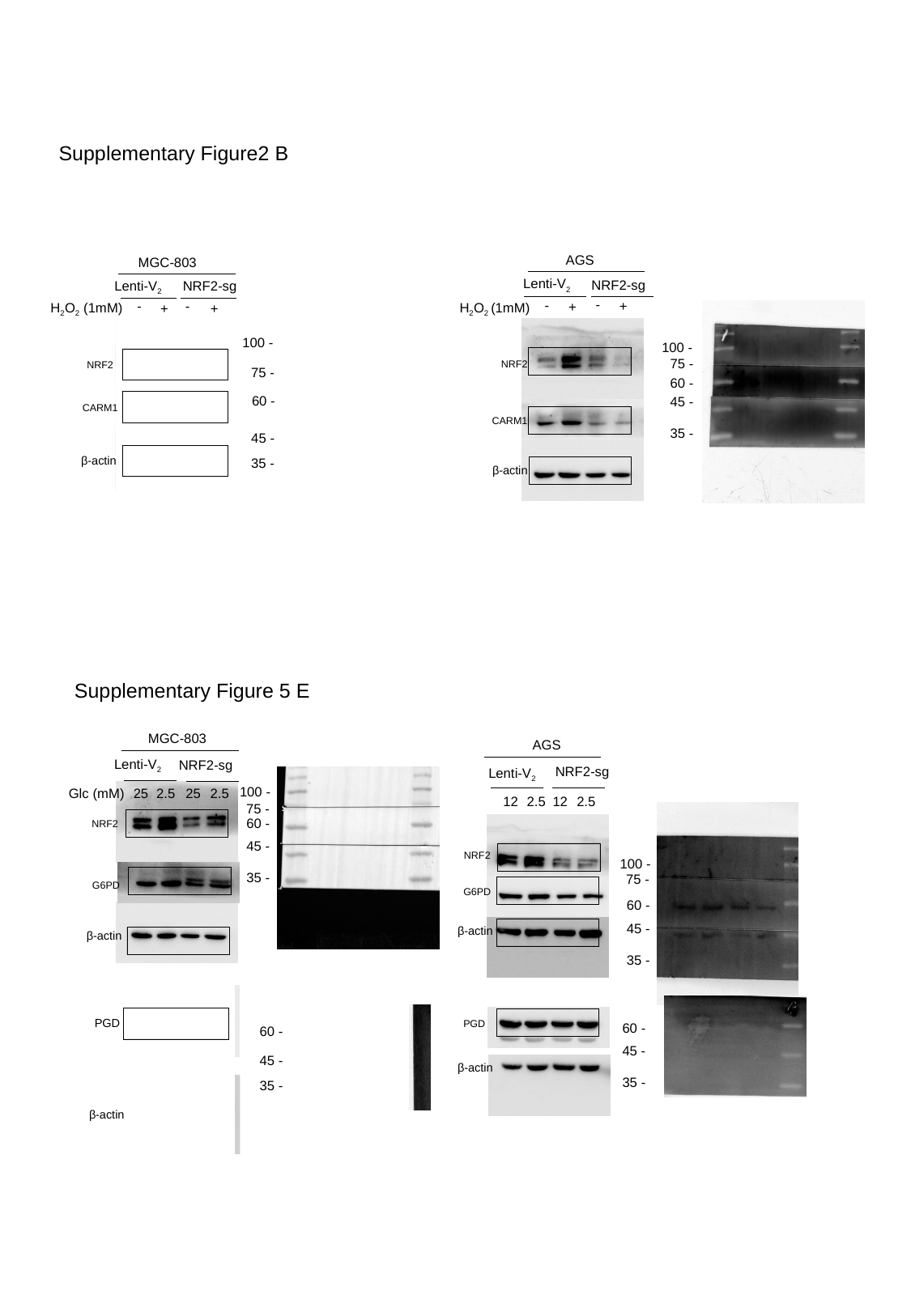

Supplementary Figure2 B
AGS
MGC-803
Lenti-V2
NRF2-sg
Lenti-V2
NRF2-sg
-
-
+
-
-
+
H2O2 (1mM)
H2O2 (1mM)
+
+
100 -
75 -
60 -
45 -
35 -
100 -
75 -
NRF2
NRF2
60 -
45 -
CARM1
CARM1
35 -
β-actin
β-actin
Supplementary Figure 5 E
MGC-803
AGS
Lenti-V2
NRF2-sg
NRF2-sg
Lenti-V2
100 -
60 -
45 -
35 -
75 -
Glc (mM)
25
2.5
25
2.5
12
2.5
12
2.5
NRF2
NRF2
100 -
75 -
G6PD
G6PD
60 -
45 -
β-actin
β-actin
35 -
PGD
PGD
60 -
60 -
45 -
35 -
45 -
β-actin
35 -
β-actin

## Slide 7
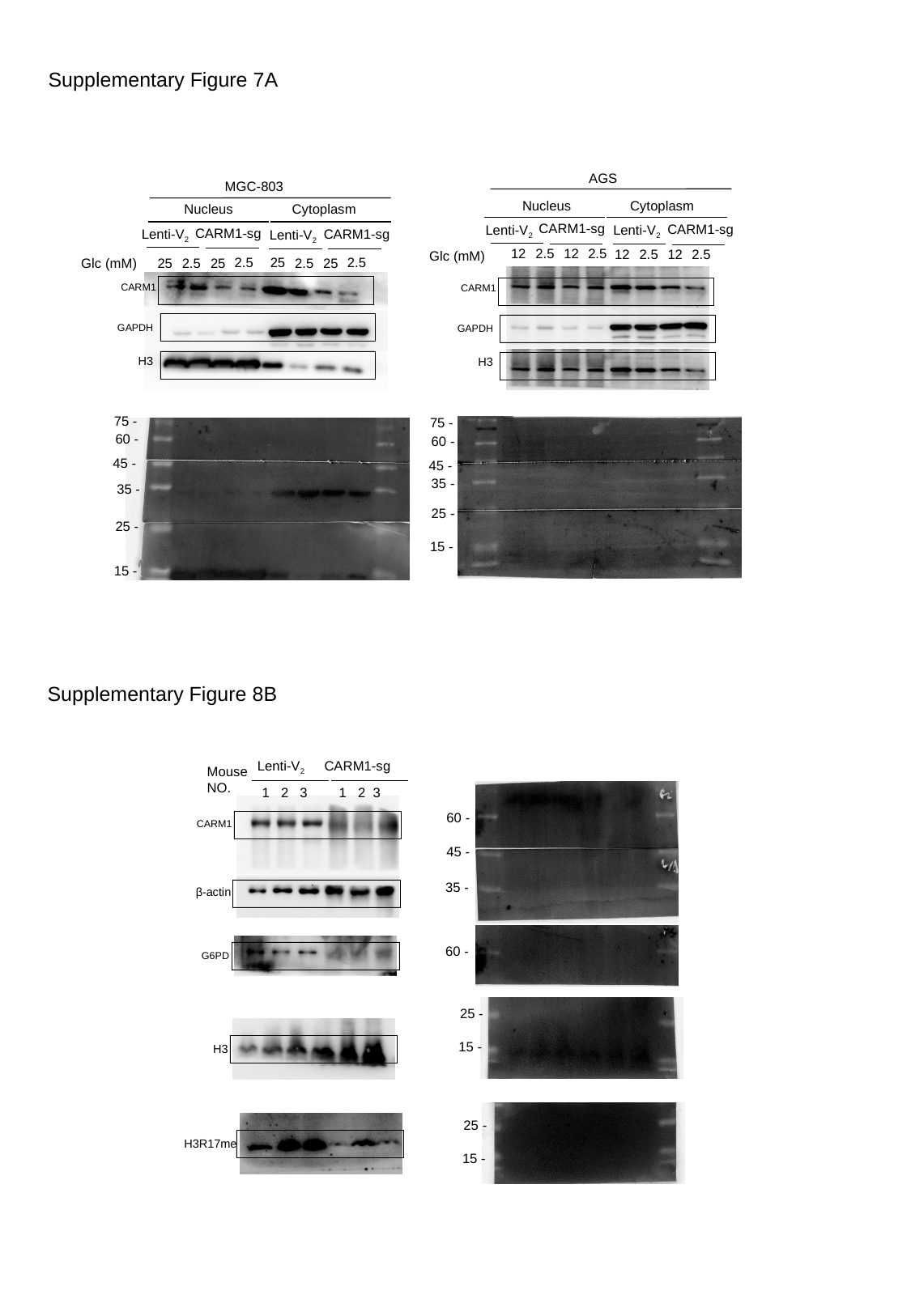

Supplementary Figure 7A
AGS
MGC-803
Nucleus
Cytoplasm
Nucleus
Cytoplasm
CARM1-sg
CARM1-sg
Lenti-V2
Lenti-V2
CARM1-sg
CARM1-sg
Lenti-V2
Lenti-V2
12
2.5
12
2.5
12
2.5
12
2.5
Glc (mM)
2.5
2.5
25
25
2.5
25
Glc (mM)
2.5
25
CARM1
CARM1
GAPDH
GAPDH
H3
H3
75 -
75 -
60 -
60 -
45 -
45 -
35 -
35 -
25 -
25 -
15 -
15 -
Supplementary Figure 8B
Lenti-V2
CARM1-sg
Mouse NO.
1 2 3
1 2 3
60 -
45 -
35 -
60 -
CARM1
β-actin
G6PD
25 -
15 -
H3
25 -
H3R17me
15 -
